# Supplementary material for: Discovery of novel TACE inhibitors using graph convolutional network, molecular docking, molecular dynamics simulation, and Biological evaluation
Source: PLoS One. 2024 Dec 27;19(12):e0315245. doi: 10.1371/journal.pone.0315245 (PMC11676921; doi:10.1371/journal.pone.0315245)
Supplement: S1 Table — (DOCX) [file pone.0315245.s001.docx]

Supplementary Data

Discovery of Novel TACE Inhibitors Using Graph Convolutional Network, Molecular Docking, Molecular Dynamics Simulation, and Biological Evaluation

Muhammad Yasir^1^, Jinyoung Park^1^, Eun-Taek Han^2^, Jin-Hee Han^2^, Won Sun Park^3^,

Mubashir Hassan^4^, Andrzej Kloczkowski^4^, Wanjoo Chun^1,*^

^1^Department of Pharmacology, Kangwon National University School of Medicine, Chuncheon, 24341, Republic of Korea;

^2^Department of Medical Environmental Biology and Tropical Medicine, Kangwon National University School of Medicine, Chuncheon, 24341, Republic of Korea;

^3^Department of Physiology, Kangwon National University School of Medicine, Chuncheon, 24341, Republic of Korea;

^4^The Steve and Cindy Rasmussen Institute for Genomic Medicine at Nationwide Children's Hospital, Columbus, OH 43205, USA

**Corresponding author**: Dr. Wanjoo Chun, Department of Pharmacology Kangwon National University School of Medicine, Kangwon National University, Email: [wchun@kangwon.ac.kr](mailto:wchun@kangwon.ac.kr), Phone: +82-33-250-8853.

**S1 Table.** Docking energy score of all 33 docked compounds.

| **Sr no** | **Compounds** | **CDocker energy**  **(kcal/mol)** | **CDocker interaction energy**  **(kcal/mol)** |
| --- | --- | --- | --- |
| 1 | Quinapril | -63.0703 | -79.1213 |
| 2 | Vorinostat | -54.7973 | -60.0783 |
| 3 | Alvimopan | -52.7959 | -65.4044 |
| 4 | Marimastat | -49.1011 | -57.4456 |
| 5 | BMS-561392 | -46.8811 | -84.7487 |
| 6 | Bufexamac | -44.9771 | -51.3456 |
| 7 | Loxoprofen | -44.2524 | -58.5632 |
| 8 | Thiamine | -41.7703 | -45.0779 |
| 9 | Adrafinil | -41.099 | -50.5074 |
| 10 | Naproxen | -37.2369 | -53.9294 |
| 11 | Phenformin | -36.9916 | -41.0075 |
| 12 | Zinc_Acetate | -35.5984 | -33.0282 |
| 13 | Cetirizine | -34.6891 | -54.0816 |
| 14 | Liarozole | -32.084 | -45.1788 |
| 15 | Detomidine | -31.7856 | -34.8698 |
| 16 | Ondansetron | -31.3707 | -47.4873 |
| 17 | Tipiracil | -30.7788 | -42.859 |
| 18 | Phenytoin | -29.7108 | -38.4744 |
| 19 | Alosetron | -29.6526 | -45.9464 |
| 20 | Carboplatin | -25.6195 | -38.1262 |
| 21 | Xylometazoline | -23.9756 | -36.0702 |
| 22 | Pramipexole | -23.6138 | -33.7614 |
| 23 | Moroxydine | -22.9465 | -30.851 |
| 24 | Diphenylamine | -22.0168 | -29.2102 |
| 25 | Tolazoline | -18.8227 | -33.1966 |
| 26 | Benzamidine | -18.3724 | -24.1218 |
| 27 | Ligustrazine | -17.3682 | -24.5747 |
| 28 | Doxapram | -11.9835 | -35.8748 |
| 29 | Naphazoline | -11.4567 | -37.3213 |
| 30 | Tofogliflozin | -5.045 | -59.4146 |
| 31 | Triprolidine | 1.07384 | -39.4733 |
| 32 | Quinine | 14.8476 | -51.0632 |
| 33 | Varenicline | 16.5849 | -28.949 |
