# Supplementary material for: Discovery of novel TACE inhibitors using graph convolutional network, molecular docking, molecular dynamics simulation, and Biological evaluation
Source: PLoS One. 2024 Dec 27;19(12):e0315245. doi: 10.1371/journal.pone.0315245 (PMC11676921; doi:10.1371/journal.pone.0315245)
Supplement: Attachment — Submitted filename: PONE-D-24-43186.pdf [file pone.0315245.s002.pdf]

PLOS ONE

Discovery of Novel TACE Inhibitors Using Graph Convolutional Network, Molecular Docking, Molecular Dynamics Simulation, and Biological Evaluation

--Manuscript Draft--

**Manuscript Number:**	PONE-D-24-43186
**Article Type:**	Research Article
**Full Title:**	Discovery of Novel TACE Inhibitors Using Graph Convolutional Network, Molecular Docking, Molecular Dynamics Simulation, and Biological Evaluation
**Short Title:**	Computational investigation of Novel TACE inhibitors
**Corresponding Author:**	wanjoo chun<br>Kangwon National University School of Medicine<br>Chuncheon, KOREA, REPUBLIC OF
**Keywords:**	TNF-α converting enzyme (TACE); Vorinostat; DeepChem; GraphConvMol; Molecular Docking; Molecular dynamics (MD) simulation; RAW 264.7 cells
**Abstract:**	The increasing utilization of deep learning models in drug repositioning has proven to be highly efficient and effective. In this study, we employed an integrated deep-learning model followed by traditional drug screening approach to screen a library of FDA-approved drugs, aiming to identify novel inhibitors targeting the TNF-α converting enzyme (TACE). TACE, also known as ADAM17, plays a crucial role in the inflammatory response by converting pro-TNF-α to its active soluble form and cleaving other inflammatory mediators, making it a promising target for therapeutic intervention in diseases such as rheumatoid arthritis. Reference datasets containing active and decoy compounds specific to TACE were obtained from the DUD-E database. Using RDKit, a cheminformatics toolkit, we extracted molecular features from these compounds. We applied the GraphConvMol model within the DeepChem framework, which utilizes graph convolutional networks, to build a predictive model based on the DUD-E datasets. Our trained model was subsequently used to predict the TACE inhibitory potential of FDA-approved drugs. From these predictions, Vorinostat was identified as a potential TACE inhibitor. Moreover, molecular docking and molecular dynamics simulation were conducted to validate these findings, using BMS-561392 as a reference TACE inhibitor. Vorinostat, originally an FDA-approved drug for cancer treatment, exhibited strong binding interactions with key TACE residues, suggesting its repurposing potential. Biological evaluation with RAW 264.7 cell confirmed the computational results, demonstrating that Vorinostat exhibited comparable inhibitory activity against TACE. In conclusion, our study highlights the capability of deep learning models to enhance virtual screening efforts in drug discovery, efficiently identifying potential candidates for specific targets such as TACE. Vorinostat, as a newly identified TACE inhibitor, holds promise for further exploration and investigation in the treatment of inflammatory diseases like rheumatoid arthritis.
**Order of Authors:**	Muhammad Yasir
	Jinyoung Park
	Eun-Taek Han
	Jin-Hee Han
	Won Sun Park
	Mubashir Hassan
	Andrzej Kloczkowski
	wanjoo chun
**Additional Information:**	
**Question**	**Response**
**Financial Disclosure**	Yes

Powered by Editorial Manager® and ProduXion Manager® from Aries Systems Corporation

Enter a financial disclosure statement that describes the sources of funding for the work included in this submission. Review the submission guidelines for detailed requirements. View published research articles from *PLOS ONE* for specific examples.<br><br>This statement is required for submission and **will appear in the published article** if the submission is accepted. Please make sure it is accurate.<br><br>**Funded studies**<br>Enter a statement with the following details:<br>• Initials of the authors who received each award<br>• Grant numbers awarded to each author<br>• The full name of each funder<br>• URL of each funder website<br>• Did the sponsors or funders play any role in the study design, data collection and analysis, decision to publish, or preparation of the manuscript?<br><br>Did you receive funding for this work?	
Please add funding details.<br>as follow-up to "**Financial Disclosure**<br><br>Enter a financial disclosure statement that describes the sources of funding for the work included in this submission. Review the submission guidelines for detailed requirements. View published research articles from *PLOS ONE* for specific examples.<br><br>This statement is required for submission and **will appear in the published article** if the submission is accepted. Please make sure it is accurate.	This work was supported by a Research Grant from the Institute of Medical Sciences, Kangwon National University 2024, a Korea Basic Science Institute (National Research Facilities and Equipment Center) grant funded by the Ministry of Education (grant no. 2022R1A6C101A739), and NIH grant numbers R01GM127701 and R01HG012117.

Powered by Editorial Manager® and ProduXion Manager® from Aries Systems Corporation

**Funded studies**<br>Enter a statement with the following details:<br>• Initials of the authors who received each award<br>• Grant numbers awarded to each author<br>• The full name of each funder<br>• URL of each funder website<br>• Did the sponsors or funders play any role in the study design, data collection and analysis, decision to publish, or preparation of the manuscript?<br><br>Did you receive funding for this work?"	
Please select the country of your main research funder (please select carefully as in some cases this is used in fee calculation).<br>as follow-up to "**Financial Disclosure**<br><br>Enter a financial disclosure statement that describes the sources of funding for the work included in this submission. Review the submission guidelines for detailed requirements. View published research articles from *PLOS ONE* for specific examples.<br><br>This statement is required for submission and **will appear in the published article** if the submission is accepted. Please make sure it is accurate.<br><br>**Funded studies**<br>Enter a statement with the following details:<br>• Initials of the authors who received each award<br>• Grant numbers awarded to each author<br>• The full name of each funder<br>• URL of each funder website<br>• Did the sponsors or funders play any role in the study design, data collection and analysis, decision to publish, or preparation of the manuscript?<br><br>Did you receive funding for this work?"	KOREA, REPUBLIC OF - KR
**Competing Interests**	The authors have declared that no competing interests exist.

Use the instructions below to enter a competing interest statement for this submission. On behalf of all authors, disclose any competing interests that could be perceived to bias this work—acknowledging all financial support and any other relevant financial or non-financial competing interests.<br><br>This statement is required for submission and **will appear in the published article** if the submission is accepted. Please make sure it is accurate and that any funding sources listed in your Funding Information later in the submission form are also declared in your Financial Disclosure statement.<br><br>View published research articles from *PLOS ONE* for specific examples.<br><br>**NO authors have competing interests**<br><br>Enter: *The authors have declared that no competing interests exist.*<br><br>**Authors with competing interests**<br><br>Enter competing interest details beginning with this statement:<br><br>*I have read the journal's policy and the authors of this manuscript have the following competing interests: [insert competing interests here]*<br><br>* typeset	
**Ethics Statement**<br><br>Enter an ethics statement for this submission. This statement is required if the study involved:<br><br>• Human participants<br>• Human specimens or tissue<br>• Vertebrate animals or cephalopods<br>• Vertebrate embryos or tissues	N/A

• Field research<br><br>Write "N/A" if the submission does not require an ethics statement.<br><br>General guidance is provided below. Consult the submission guidelines for detailed instructions. **Make sure that all information entered here is included in the Methods section of the manuscript.**	

Powered by Editorial Manager® and ProduXion Manager® from Aries Systems Corporation

**Format for specific study types**<br><br>**Human Subject Research (involving human participants and/or tissue)**<br>• Give the name of the institutional review board or ethics committee that approved the study<br>• Include the approval number and/or a statement indicating approval of this research<br>• Indicate the form of consent obtained (written/oral) or the reason that consent was not obtained (e.g. the data were analyzed anonymously)<br><br>**Animal Research (involving vertebrate animals, embryos or tissues)**<br>• Provide the name of the Institutional Animal Care and Use Committee (IACUC) or other relevant ethics board that reviewed the study protocol, and indicate whether they approved this research or granted a formal waiver of ethical approval<br>• Include an approval number if one was obtained<br>• If the study involved *non-human primates*, add *additional details* about animal welfare and steps taken to ameliorate suffering<br>• If anesthesia, euthanasia, or any kind of animal sacrifice is part of the study, include briefly which substances and/or methods were applied<br><br>**Field Research**<br><br>Include the following details if this study involves the collection of plant, animal, or other materials from a natural setting:<br>• Field permit number<br>• Name of the institution or relevant body that granted permission	
**Data Availability**<br><br>Authors are required to make all data underlying the findings described fully available, without restriction, and from the time of publication. PLOS allows rare exceptions to address legal and ethical concerns. See the PLOS Data Policy and FAQ for detailed information.	Yes - all data are fully available without restriction

Powered by Editorial Manager® and ProduXion Manager® from Aries Systems Corporation

A Data Availability Statement describing where the data can be found is required at submission. Your answers to this question constitute the Data Availability Statement and **will be published in the article**, if accepted.<br><br>**Important:** Stating 'data available on request from the author' is not sufficient. If your data are only available upon request, select 'No' for the first question and explain your exceptional situation in the text box.<br><br>Do the authors confirm that all data underlying the findings described in their manuscript are fully available without restriction?	
**Describe where the data may be found in full sentences. If you are copying our sample text, replace any instances of XXX with the appropriate details.**<br><br>• If the data are **held or will be held in a public repository**, include URLs, accession numbers or DOIs. If this information will only be available after acceptance, indicate this by ticking the box below. For example: *All XXX files are available from the XXX database (accession number(s) XXX, XXX.*).<br>• If the data are all contained **within the manuscript and/or Supporting Information files**, enter the following: *All relevant data are within the manuscript and its Supporting Information files.*<br>• If neither of these applies but you are able to provide **details of access elsewhere**, with or without limitations, please do so. For example:<br><br>*Data cannot be shared publicly because of [XXX]. Data are available from the XXX Institutional Data Access / Ethics Committee (contact via XXX) for researchers who meet the criteria for access to confidential data.*<br><br>*The data underlying the results presented in the study are available from (include the name of the third party*	All relevant data are within the manuscript and its Supporting Information files.

and contact information or URL).<br>• This text is appropriate if the data are owned by a third party and authors do not have permission to share the data.<br><br>* typeset	
Additional data availability information:	

Manuscript

Click here to access/download;Manuscript;Yasir_Manuscript.docx

Discovery of Novel TACE Inhibitors Using Graph Convolutional Network, Molecular Docking, Molecular Dynamics Simulation, and Biological Evaluation

Muhammad Yasir[1], Jinyoung Park[1], Eun-Taek Han[2], Jin-Hee Han[2], Won Sun Park[3], Mubashir Hassan[4], Andrzej Kloczkowski[4], Wanjoo Chun[1,*]

[1]Department of Pharmacology, Kangwon National University School of Medicine, Chuncheon, 24341, Republic of Korea;

[2]Department of Medical Environmental Biology and Tropical Medicine, Kangwon National University School of Medicine, Chuncheon, 24341, Republic of Korea;

[3]Department of Physiology, Kangwon National University School of Medicine, Chuncheon, 24341, Republic of Korea;

[4]The Steve and Cindy Rasmussen Institute for Genomic Medicine at Nationwide Children's Hospital, Columbus, OH 43205, USA

Corresponding author: Dr. Wanjoo Chun, Department of Pharmacology Kangwon National University School of Medicine, Kangwon National University, Email: wchun@kangwon.ac.kr, Phone: +82-33-250-8853.

Abstract

The increasing utilization of deep learning models in drug repositioning has proven to be highly efficient and effective. In this study, we employed an integrated deep-learning model followed by traditional drug screening approach to screen a library of FDA-approved drugs, aiming to identify novel inhibitors targeting the TNF-α converting enzyme (TACE). TACE, also known as ADAM17, plays a crucial role in the inflammatory response by converting pro-TNF-α to its active soluble form and cleaving other inflammatory mediators, making it a promising target for therapeutic intervention in diseases such as rheumatoid arthritis. Reference datasets containing active and decoy compounds specific to TACE were obtained from the DUD-E database. Using RDKit, a cheminformatics toolkit, we extracted molecular features from these compounds. We applied the GraphConvMol model within the DeepChem framework, which utilizes graph convolutional networks, to build a predictive model based on the DUD-E datasets. Our trained model was subsequently used to predict the TACE inhibitory potential of FDA-approved drugs. From these predictions, Vorinostat was identified as a potential TACE inhibitor. Moreover, molecular docking and molecular dynamics simulation were conducted to validate these findings, using BMS-561392 as a reference TACE inhibitor. Vorinostat, originally an FDA-approved drug for cancer treatment, exhibited strong binding interactions with key TACE residues, suggesting its repurposing potential. Biological evaluation with RAW 264.7 cell confirmed the computational results, demonstrating that Vorinostat exhibited comparable inhibitory activity against TACE. In conclusion, our study highlights the capability of deep learning models to enhance virtual screening efforts in drug discovery, efficiently identifying potential candidates for specific targets such as TACE. Vorinostat, as a newly identified TACE inhibitor, holds promise for further exploration and investigation in the treatment of inflammatory diseases like rheumatoid arthritis.

Keywords: TNF-α converting enzyme (TACE); Vorinostat; DeepChem; GraphConvMol; Molecular docking; Molecular dynamics (MD) simulation; RAW 264.7 cells

Introduction

Tumor necrosis factor-alpha (TNF-α) plays a pivotal role in the inflammatory response and is a key cytokine involved in the pathogenesis of various inflammatory diseases, including rheumatoid arthritis [1, 2]. The biological activities of TNF-α extend beyond inflammation to include the regulation of immune cells, apoptosis, and inhibition of tumorigenesis, highlighting its dual role in health and disease [3-6]. Understanding the regulation and function of TNF-α is essential for developing therapeutic strategies aimed at controlling inflammatory conditions. TNF-α is synthesized as a 26 kDa transmembrane precursor, known as pro-TNF-α. This precursor form undergoes a proteolytic cleavage to produce a 17 kDa soluble mature protein, a process catalyzed by the TNF-α converting enzyme (TACE) [7, 8]. TACE, also known as a disintegrin and metalloproteinase 17 (ADAM17), is a membrane-bound enzyme that plays a crucial role in the conversion of pro-TNF-α to its active soluble form [9-11].

The activity of TACE is not limited to TNF-α; it also cleaves a variety of other cell surface proteins. The broad substrate specificity of TACE underscores its significant role in modulating various physiological and pathological processes. By cleaving cytokines and their receptors, TACE influences cytokine signaling pathways, which are crucial for immune responses and inflammation. For instance, the cleavage of IL-6R by TACE leads to the activation of IL-6R-mediated signal transduction, contributing to the inflammatory cascade [12, 13]. Similarly, TACE-mediated cleavage of TNF-R influences TNFR-mediated signaling pathways, which are vital for immune regulation and inflammatory responses [14, 15]. The modulation of these pathways by TACE suggests that its activity is a critical pro-inflammatory event. The role of TACE in inflammation is further exemplified by its involvement in the shedding of ligands of the ErbB receptor family. ErbB receptors are implicated in cell growth, differentiation, and survival, and their ligands, such as TGFα and amphiregulin, are essential for the activation of these receptors. TACE-mediated cleavage of these ligands affects ErbB signaling pathways, which have been associated with inflammation and tissue remodeling [16]. Additionally, the cleavage of adhesion molecules like ICAM-1 by TACE modulates leukocyte adhesion and migration, processes that are fundamental to the inflammatory response [17]. Furthermore,

TNF-α, is also produced by neurons and glia, plays a significant role by promoting inflammatory responses and possibly inducing Aβ production in neuroinflammations. TACE is crucial in this process, converting precursor TNF-α to its soluble form and cleaving TNFR1 and TNFR2 to their soluble counterparts. Elevated levels of TNF-α, sTNFR1, and sTNFR2 in the cerebrospinal fluid and plasma are associated with Alzheimer's disease (AD) and mild cognitive impairment (MCI), and higher TACE activity has been observed in both MCI and AD patients. This underscores the importance of TNF-α and TACE in the inflammatory pathology of AD [18, 19].

Given the central role of TACE in converting pro-TNF-α to its soluble form and its involvement in the regulation of other inflammatory mediators, TACE has emerged as a potential therapeutic target for inflammatory diseases [18, 20]. The inhibition of TACE activity could potentially reduce the levels of soluble TNF-α and other pro-inflammatory molecules, thereby mitigating inflammation and its associated pathologies. Several studies have focused on developing TACE inhibitors as therapeutic agents for conditions such as rheumatoid arthritis, psoriasis, and other inflammatory disorders [9, 21-23]. These inhibitors aim to block the proteolytic activity of TACE, preventing the release of soluble TNF-α and other substrates, and consequently dampening the inflammatory response.

Recently, the integration of deep learning models in the drug discovery process has gained significant attention Therefore, its potential to identify novel TACE inhibitors with greater accuracy and efficiency was examined in this study. These models leverage large datasets and algorithms to screen the compounds with potential binding affinities and activities, thus streamlining the discovery of effective therapeutic candidates. By incorporating deep learning into traditional drug screening, researchers can rapidly screen vast libraries of compounds to identify promising drug candidates with high reliability, leading to further biological validations and trials therefore, fasten the drug discovery process.

Materials and Methods

TACE dataset, FDA-approved drug library, and commercial Enamine compound library

The DUD-E website provided access to datasets containing TACE active and decoy compounds (https://dude.docking.org/). The active dataset included 532 compounds, while the decoy dataset comprised 35,900 compounds. Each molecule was represented by canonical SMILES strings, accompanied by corresponding DUD-E ID and ChEMBL ID numbers, with the legend distinguishing between active and decoy compounds. Additionally, an FDA-approved drug library consisting of 3,105 compounds was obtained from the Selleck Chemicals website (https://www.selleckchem.com). Initially formatted in SDF (structure-data file), the FDA-approved drugs were converted into SMILES strings using RDKit. Furthermore, BMS-561392, a known TACE inhibitor, was obtained from MedChemExpress (https://www.medchemexpress.com/) in SMILES format, serving as a reference compound.

Please state the date of access , because after your access, more data could have been deposited

Molecular descriptor generation using RDKit

Molecular descriptors for all the compounds were generated using RDKit

For extracting molecular descriptors from the compounds, we utilized RDKit, an open-source, high-performance cheminformatics and machine learning toolkit available at https://www.rdkit.org. This toolkit is implemented in Python and provides functionalities for computing molecular descriptors, creating chemical features, and visualizing chemical data.

was

Deep learning architecture

The TACE active and decoy datasets were split into training, validation, and test sets using an 8:1:1 ratio. Following this division, we conducted deep learning evaluations using the GraphConvModel from DeepChem (https://deepchem.io/models). GraphConvModel, a type of graph convolutional neural network, is particularly adept at learning features from graph-structured input data such as molecular graphs. This model preprocesses molecular structures into graphs, where atoms represent nodes and bonds represent edges. Node features are extracted using the ConvMolFeaturizer, which generates atom-level features such as atom type, formal charge, chirality, degree, number of hydrogens, and hybridization state. Edge features correspond to the adjacency list of the molecule, indicating the connectivity between atoms. The model includes two graph convolutional layers with 64 hidden units each, followed by batch normalization and ReLU activation to stabilize and accelerate training while introducing non-linearity. A dense layer with 128 units is added after the graph convolutional layers, with

Please justify the use of the deep learning architecture by making references to some other sources

a dropout rate of 0.25 to prevent overfitting. The readout layer aggregates node features to produce a graph-level representation, and the final output layer is configured for binary classification with a single output. The model uses a batch size of 128 and saves the trained model. For the dataset, the RandomSplitter is employed to split the dataset, and the model's performance is evaluated using the Matthews correlation coefficient (MCC) as the primary metric.

Please rephrase this as the statement is not clear

Throughout the training process, the model works to minimize the loss function concerning the input molecular datasets. By employing backpropagation, the convolutional layer weights are adjusted to optimize the prediction of properties such as solubility, bioactivity, and toxicity

Has this approach been tried before

based on the provided molecular structures. This architecture effectively captures the relational information between atoms in molecules, enabling accurate predictions for molecular property prediction and drug discovery tasks. Detailed information on the model's architecture was provided as a Colab Jupyter Notebook file on a GitHub repository (https://github.com/Yasirubunt/yasirknu.github.io).

Accession of TACE crystalized structure

The three-dimensional structure of the TACE, identified by the PDB ID: 2OI0 with a resolution of 2.00Å, was acquired from the Protein Data Bank (PDB) accessible at https://www.rcsb.org/. The TACE crystal structure, comprising alpha-helices, beta-sheets, coils, and turns, underwent a comprehensive quantitative structural analysis utilizing the VADAR internet server (http://vadar.wishartlab.com/). Subsequently, energy minimization and Ramachandran Plot analysis were carried out utilizing UCSF Chimera and Discovery Studio [24, 25].

Prediction of active binding site

The positioning of a ligand within a protein's holo-structure is a key determinant of the protein's binding pocket [26]. The TACE and co-crystallized ligand, already available on PDB (PDB ID: 2OI0), was further utilized for binding pocket analysis. The identification of interacting amino acids was accomplished through Discovery Studio's ligand interaction approach, ensuring precision in the generation of the binding site. Additionally, the binding pocket residues were confirmed from already published data [27]. Therefore, the bound ligand was chosen by ligand

selection, and the binding sphere was created using the current selection approach within the defined Binding Site window of Discovery Studio. Subsequently, the binding sphere was reduced with imposed restrictions specific to the selected amino acids for the accuracy and precision of docking.

Molecular Docking

Molecular docking stands as the extensively employed approach for assessing the interactions between ligands and receptors [28]. It forecasts the strength of association or binding energy of protein-ligand complexes by assessing their preferred orientations, using scoring algorithms in the process [29]. The already bound ligand molecule and water molecules were deleted from the protein. The Zn2+ ion was retained in its native position to accurately represent the binding environment, as it plays a crucial role in stabilizing the protein structure and facilitating ligand binding. The coordination geometry and charge state of the Zn2+ ion were carefully maintained to ensure realistic interactions with the ligands.

what is the source of this fact?

Furthermore, the hydrogens were appended to the protein utilizing Discovery Studio's receptor preparation module. Moreover, the ligand preparations for both reference and candidate compounds included the generation of tautomers, adjustment of ionization states, and correction of any bad valences. These tasks were performed using the Ligands Preparation module in Discovery Studio. The molecular docking of ligands against the target protein (TACE) was conducted using the CDocker module in Discovery Studio, employing default orientation and conformation settings. The presence of the Zn2+ ion was reconfirmed before starting the molecular docking, ensuring that the ligands could form interactions with it where applicable. Therefore, the docked complexes were assessed based on the docking energy score kcal/mol.

Molecular Dynamics Simulations

The Molecular Dynamics (MD) simulation protocols followed were adapted from our previously published paper involving a 100 ns simulation [30], in which we screened JAK2 inhibitors. Therefore, the top seven compounds with the lowest docking energies were selected for the MD simulation, alongside BMS-561392 as a reference compound for comparative

analysis. The CHARMM-GUI web server (https://www.charmm-gui.org/?doc=input/solution) was used to generate input files for MD simulations with GROMACS [31]. TACE, Ligand, and Zn2+ ion were uploaded to the server for each complex and CHARMM36 force field [32] setup was performed using the solution builder protocol on the CHARMM-GUI web server. The system was solvated using the TIP3P in a cubic box under periodic boundary conditions [33], with neutralization achieved by adding counter ions. Electrostatic and van der Waals interactions were computed using the Verlet method with a cutoff radius of 10, and bond lengths were maintained using the LINCS algorithm during simulations. The Particle Mesh Ewald (PME) method was employed for accurate long-range electrostatic calculations, providing precision in the modeling of interactions involving the Zn2+ ion and surrounding residues.

Energy minimization using the steepest descent method prepared the solvated systems. Two equilibration phases followed, first under constant temperature and volume (NVT) conditions and then under constant temperature and pressure (NPT) conditions. Conversion of GROMACS topology (top) and parameter (itp) files for MD simulations was facilitated by a Python script from CHARMM-GUI, ensuring consistency and accuracy of the force field parameters, particularly for the Zn2+ coordination environment. At the end, structural analysis of protein-ligand complexes was conducted using GROMACS version 2019.3 on a Linux system [34], with a time step of 2fs used for the MD simulations.

Binding free energy Calculation of Simulated Compound

Predictions of binding free energy were made using an MM/PBSA approach from MD simulation trajectories in explicit solvent, examining the three components complex, receptor, and ligand individually [35]. The binding free energy (ΔGbinding) of the lead compounds in complex with the protein was calculated using the following equation:

$$\Delta G\text{binding} = G\text{complex} - (G\text{protein} + G\text{ligand}) \quad (1)$$

In this equation, Gcomplex denotes the energy of the lead compounds-protein complexes, while Gprotein and Gligand represent the energies of the proteins and ligands in an aqueous environment, respectively [47].

Reagents and Cell Culture

Bacterial lipopolysaccharide (LPS) from Escherichia coli serotype 055:B5 was purchased from Sigma-Aldrich (St. Louis, MO, USA). BMS-561392, Quinapril hydrochloride, Marimastat, and Bufexamac, were purchased from MedChemExpress (Monmouth Junction, NJ, USA). Vorinostat and Thiamine hydrochloride were purchased from Sigma-Aldrich (St. Louis, MO, USA). Raw 264.7 macrophage cells were purchased from the Korea cell line bank (KCLB, cat #40071). Raw 264.7 macrophage cells were maintained in Dulbecco's modified Eagle's medium (DMEM; BYLABS, Luscience Corporation, Korea) containing 10% heat-inactivated fetal bovine serum and 100 U/ml penicillin-streptomycin (Gibco) at 37 °C, 5 % CO2. Cells were incubated in 10 μM of various reagents for 1h and then stimulated with 1 μg/ml of LPS for 4h.

ELISA assay for Cytokine

Raw 264.7 macrophage cells were pretreated with various compounds (10 μM) for 1h and then stimulated with 1 μg/ml of LPS for 4h. TNF-α levels in culture media were quantified using enzyme-linked immunosorbent (ELISA) kits (R&D system, USA) according to the manufacturer's instructions.

Statistical analysis

All values presented in Figure 10 were expressed as the mean ± SD, derived from a minimum of three independent experiments. Statistical significance was assessed using a two-tailed Student's t-test, with a value of $p<0.05$ considered statistically significant. The double (**) asterisk denotes statistical significance at $p < 0.01$.

Results and Discussion

The discovery process of novel TACE inhibitors integrates ligand-based virtual screening with a deep learning algorithm and structure-based virtual screening using molecular docking and molecular dynamics (MD) simulations, illustrated in Figure 1. This study proceeded through six distinct stages: First, datasets were acquired from the DUD-E database. Second, target-specific datasets were prepared for the deep learning model. Third, the GraphConvMol

algorithm was configured. Fourth, TACE inhibitory activity was predicted using the trained GraphConvMol model. Fifth, candidates underwent screening via molecular docking and MD simulations. Finally, experimental validation of the candidates was conducted using an enzyme activity assay.

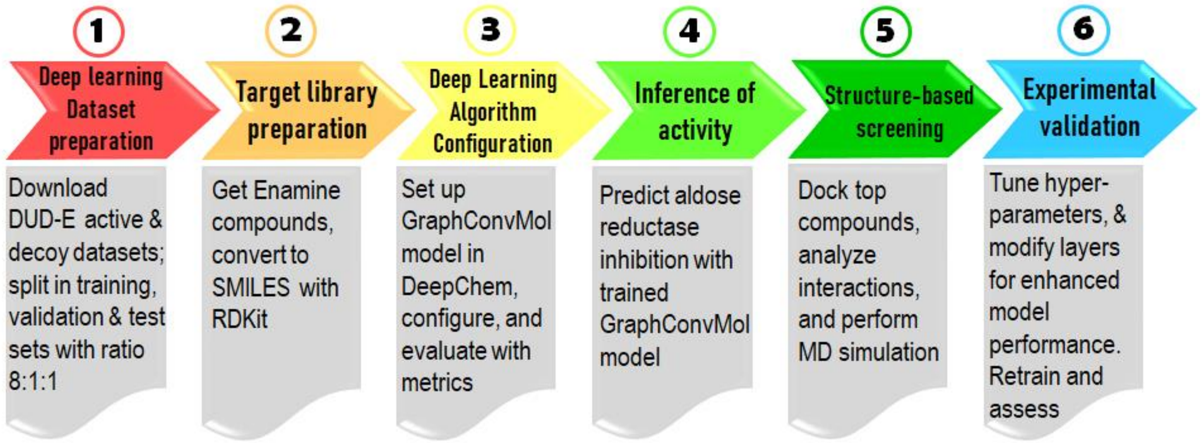

Fig 1. The workflow combines deep learning, molecular docking, MD simulations, and experimental evaluation to identify new TACE inhibitors from the pool of FDA-approved drugs.

TACE active and decoy datasets and its preprocessing using RDKit

DUD-E (Database of Useful Decoys: Enhanced) provides benchmark sets of protein-ligand complexes, featuring experimentally verified active compounds alongside decoys that lack binding affinity against various diseases. These decoys exhibit comparable physicochemical characteristics to the active compounds, yet they diverge in their two-dimensional topology [36]. The DUD-E database is extensively utilized for developing and benchmarking computational docking methods [37, 38].

The TACE dataset in the DUD-E repository (https://dude.docking.org/targets/ADA17) consists of 532 experimentally validated active compounds. Fig 2 illustrates representative structures of active and decoy compounds, with labels identifying their respective classifications. Molecular features, including molecular weight, LogP, Hbond donors, Hbond acceptors, TPSA, and rotatable bonds, were computed using RDKit, a freely available software toolkit for

This is no longer necessary

cheminformatics, to facilitate comparison of the physicochemical properties between active

and decoy compounds. No significant variance was observed in the distribution of molecular descriptor values between active and decoy compounds (Fig 3).

What is the significance of this observation and is there any literature to support the significance of this observation

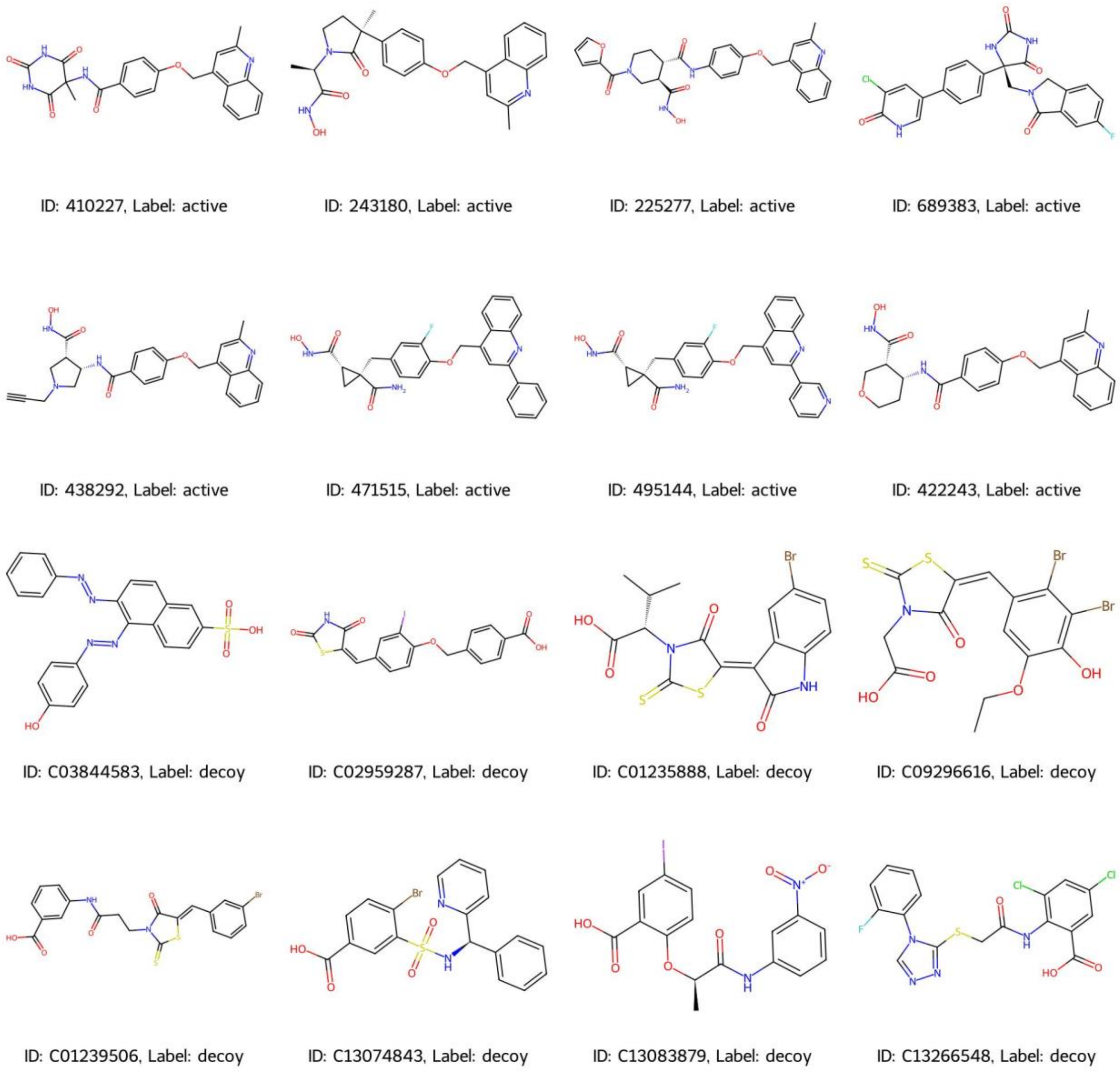

Fig 2. Illustrative depiction of active and decoy compounds.

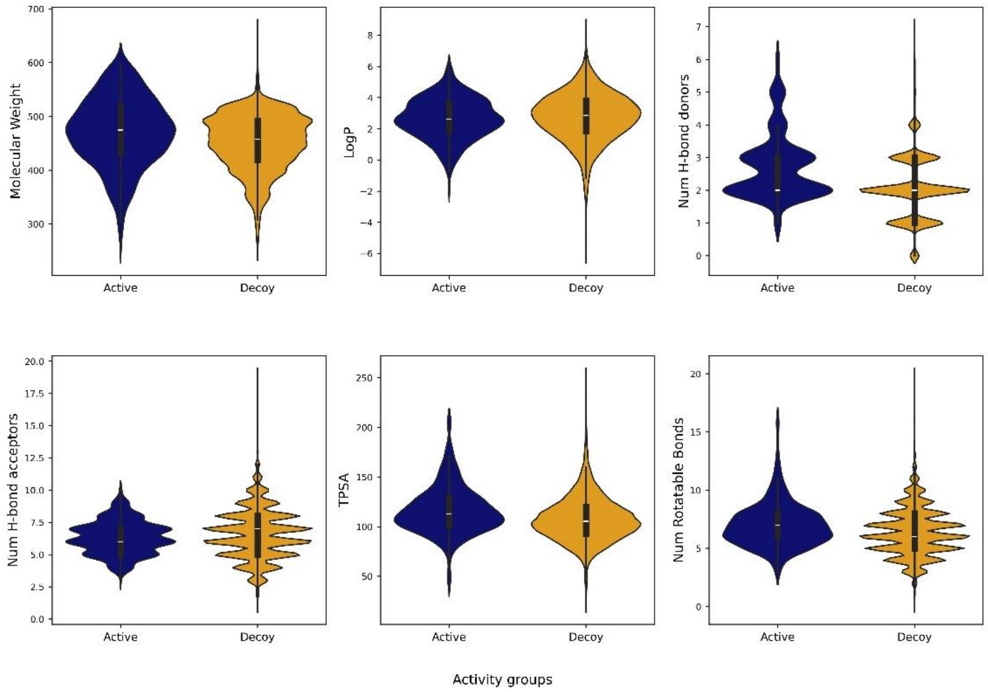

Fig 3. Distribution of molecular descriptors in active and decoy compounds.

Deep-learning model setup, training, and evaluation

DeepChem is an open-source Python library designed for applying deep learning to drug discovery and cheminformatics. This versatile library provides a comprehensive set of tools for managing molecular data. DeepChem effectively utilizes a variety of deep-learning algorithms to address tasks in drug discovery, such as predicting molecular properties, performing ligand-based virtual screening, and optimizing chemical compounds [39, 40].

In this study, the GraphConvMol model from the DeepChem library played a crucial role in distinguishing between active and decoy compounds in the TACE dataset. The GraphConvMol method comprehensively learns molecular representations, making it a powerful tool for cheminformatics applications such as predicting molecular properties and aiding in drug discovery [41, 42]. The TACE dataset was divided into training, validation, and test sets in an 8:1:1 ratio. The GraphConvMol model was then applied with 5-fold cross-validation.

To assess the model's efficacy, metrics such as the Area Under the Curve (AUC) of the Receiver Operating Characteristic (ROC) curve were calculated for the training, validation, and test datasets. The ROC curve from the 5-fold cross-validation on the training dataset demonstrated a True Positive Rate (TPR) of 1 at a very low False Positive Rate (FPR), with an

AUC value of 0.99574 (Fig 4). These results highlight the GraphConvMol model's exceptional sensitivity in accurately identifying positive instances.

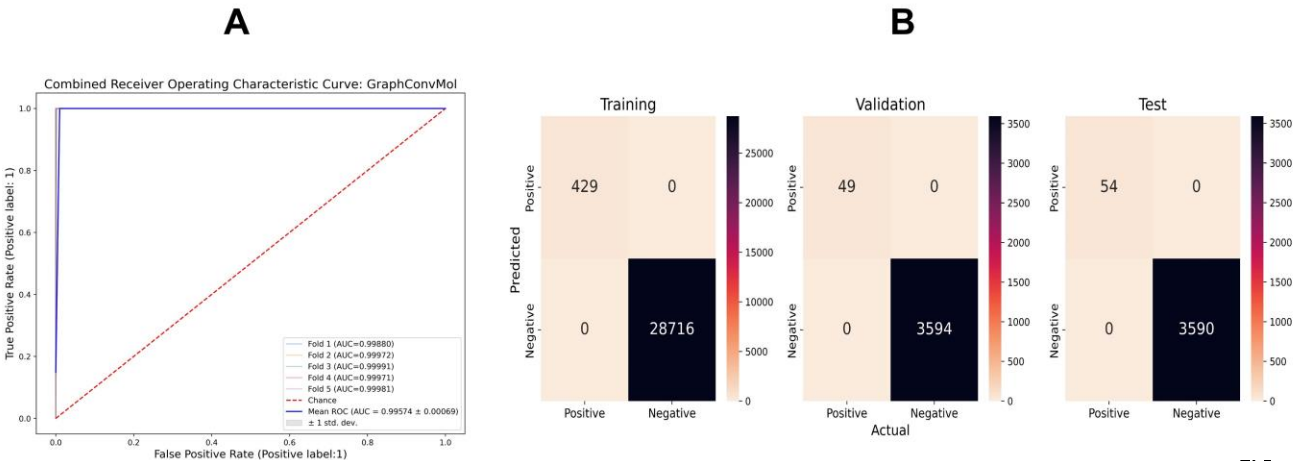

Fig 4. The AUC-ROC curve of 5-fold cross-validation of the training dataset (A) and the confusion matrix entries for the training, validation, and test datasets (B).

In the DUD-E datasets of TACE, the GraphConvMol model achieved perfect predictions, with performance metrics including precision, recall, F1 score, sensitivity, accuracy, and specificity all reaching the maximum value of 1.0. These results strongly support the model’s effectiveness in accurately identifying TACE inhibitors.

Given the imbalance in the current dataset, with significantly more decoys than active molecules, the Matthews correlation coefficient (MCC) was used to evaluate the GraphConvMol model’s efficacy due to its suitability for unbalanced datasets. The average MCC values from five-fold cross-validations were 1.00 for both the training and validation sets. An MCC of 1 indicates perfect prediction accuracy, highlighting the robustness and reliability of the model.

Prediction of TACE inhibitory potential from FDA-approved drug compound libraries

Using FDA-approved drugs to discover novel TACE inhibitors offers several advantages. Since these drugs have already undergone extensive pre-clinical and clinical testing to establish their safety, dosing, and pharmacokinetics, repurposing them can lead to faster development timelines, reduced costs, and a higher likelihood of success. In this study, an FDA-approved drug library with a unique collection of 3104 approved drugs and Active Pharmaceutical

Ingredient (API) included in pharmacopeia for high throughput screening (HTS) and high content screening (HCS), was utilized, available at https://www.selleckchem.com/screening/fda-approved-drug-library.html. Therefore, the SMILES strings of FDA-approved drugs were analyzed using the trained GraphConvMol model from DeepChem to predict their potential TACE inhibitory activity. The trained model provided predictions for the compounds, ranging from 0 (indicating no activity) to 1 (indicating high activity). Most compounds were predicted to be inactive, with only a small proportion predicted as active. Out of 3,104 FDA-approved drugs, 28 compounds were predicted to be active, with prediction values exceeding 0.90 (Table 1). For the next phase of structure-based virtual screening, we chose the top FDA-approved drugs that received high predictions from the GraphConvMol model. Additionally, we included BMS-561392, a confirmed TACE inhibitor available from MedChemExpress LLC (https://www.medchemexpress.com). These compounds were selected to be inputted into molecular docking.

Table 1. Top screened compounds from the pool of FDA library.

SMILES	Neg	Pos	Name	Target/action
C[C@H](N[C@@H](CCc1ccccc1)C(=O)O)C(=O)N1CCC[C@H]1C(=O)O.O.O	0.0009	0.9991	Enalaprilat Dihydrate	ACE inhibitor
Cl.N[C@@H](Cc1cnc[nH]1)C(=O)O.O	0.0011	0.9989	L-Histidine Hydrochloride Hydrate	Endogenous metabolite
Cl.N=C1CCCN1Cc1[nH]c(=O)[nH]c(=O)c1Cl	0.0025	0.9975	Tipiracil Hydrochloride	Thymidine phosphorylase inhibitor (TPI)
O=C(CCCCCCC(=O)Nc1ccccc1)NO	0.0047	0.9953	Vorinostat	Histone deacetylase inhibitor
CCCCOc1ccc(CC(=O)NO)cc1	0.0048	0.9952	Bufexamac	Anti-inflammatory, analgesic, antipyretic
Cc1ncc(C[n+]2csc(CCO)c2C)c(N)n1.Cl.[Cl-]	0.0050	0.9950	Thiamine Hydrochloride	Vitamin B1
CC(=O)[O-].CC(=O)[O-].O.O.[Zn+2]	0.0083	0.9917	Zinc Acetate Dihydrate	Nutritional supplement
N.N.O=C([O-])C1(C(=O)[O-])CCC1.[Pt+2]	0.0102	0.9898	Paraplatin	Antineoplastic
Cl.Cl.Cl.Cl.Nc1ccc(-c2ccc(N)c(N)c2)cc1N.O	0.0106	0.9894	3,3'-Diaminobenzidine Tetrahydrochloride Hydrate	
O=C1N=C([O-])NC1(c1ccccc1)c1ccccc1.[Na+]	0.0107	0.9893	Phenytoin	Anticonvulsant

Cl.c1ccc2c(CC3=NCCN3)cccc2c1	0.0152	0.9849	Naphazoline Hydrochloride	Alpha-adrenergic agonist
Cc1nc[nH]c1CN1CCc2c(c3ccccc3n2C)C1=O.Cl	0.0165	0.9835	Alosetron Hydrochloride	5-HT3 receptor antagonist
CNC(=O)[C@@H](NC(=O)[C@H](CC(C)C)[C@H](O)C(=O)NO)C(C)(C)C	0.0219	0.9782	Marimastat	Matrix metalloproteinase inhibitor
Cc1ncc(C[n+]2csc(CCO)c2C)c(N)n1.[Cl-]	0.0313	0.9687	Vitamin B1	Vitamin B1
C=C[C@H]1CN2CC[C@H]1C[C@H]2[C@H](O)c1ccnc2ccc(OC)cc12.Cl.O.O	0.0382	0.9618	Quinine Hydrochloride Dihydrate	Antimalarial
Cl.N=C(N)NC(=N)NCCc1ccccc1	0.0387	0.9613	Phenformin	Anti-diabetic
Cc1cccc(Cc2c[nH]cn2)c1C.Cl	0.0415	0.9585	Detomidine Hydrochloride	Alpha-adrenergic agonist
Cc1nccn1CC1CCc2c(c3ccccc3n2C)C1=O.Cl.O.O	0.0451	0.9549	Ondansetron Hydrochloride Dihydrate	Antiemetic, 5-HT3 receptor antagonist
CCc1ccc(Cc2ccc3c(c2)[C@]2(OC3)O[C@H](CO)[C@@H](O)[C@H](O)[C@H]2O)cc1.O	0.0460	0.9540	Tofogliflozin Hydrate	SGLT2 inhibitor
CCN1CC(CCN2CCOCC2)C(c2ccccc2)(c2ccccc2)C1=O.Cl.O	0.0486	0.9515	Doxapram Hydrochloride Monohydrate	CNS stimulant
C[C@H](O)C(=O)[O-].[Na+]	0.0544	0.9456	Sodium L-Lactate	Antimicrobial
Cl.c1ccc(CC2=NCCN2)cc1	0.0552	0.9449	Tolazoline Hydrochloride	Alpha-adrenergic antagonist
Cl.N=C(N)c1ccccc1	0.0659	0.9341	Benzamidine Hydrochloride	Serine protease inhibitor
Cl.Cl.c1cnc2cc3c(cc2n1)C1CNCC3C1	0.0798	0.9202	Varenicline Dihydrochloride	Nicotinic acetylcholine receptor agonist
CCCN[C@H]1CCc2nc(N)sc2C1.Cl.Cl.O	0.0804	0.9196	Pramipexole Dihydrochloride Monohydrate	Antiparkinsonian
CC(=O)[O-].[Na+]	0.0812	0.9188	Sodium Acetate	Buffering agent
CC(C(=O)[O-])c1ccc(CC2CCCC2=O)cc1.[Na+]	0.0818	0.9182	Loxoprofen Sodium	Anti-inflammatory, analgesic, antipyretic
Cc1cc(C(C)(C)C)cc(C)c1CC1=NCCN1.Cl	0.0887	0.9113	Xylometazoline Hydrochloride	H1 histamine receptor antagonist
Cc1nc(C)c(C)nc1C.Cl	0.1108	0.8892	Ligustrazine Hydrochloride	Antioxidant, anti-inflammatory
Cl.c1ccc(Nc2ccccc2)cc1	0.1120	0.8881	Diphenylamine Hydrochloride	H1 histamine receptor antagonist
Cc1ccc(/C(=C\CN2CCCC2)c2ccccn2)cc1.Cl.O	0.1178	0.8822	Triprolidine HCL	H1 histamine receptor antagonist

C[C@H]1CN(C[C@H](Cc2ccccc2)C(=O)NCC(=O)O)CC[C@@]1(C)c1cccc(O)c1.O.O	0.1203	0.8797	Alvimopan Dihydrate	µ-Opioid receptor antagonist
Cl.N=C(N)NC(=N)N1CCOCC1	0.1289	0.8711	Moroxydine Hydrochloride	Antiviral
O=C(CS(=O)C(c1ccccc1)c1ccccc1)NO	0.1298	0.8702	Adrafinil	Alpha-adrenergic agonist
COc1ccc2cc([C@H](C)C(=O)[O-])ccc2c1.[Na+]	0.1584	0.8416	Naproxen Sodium	Cyclooxygenase inhibitor
Cc1ccc(S(=O)(=O)O)cc1.O	0.1611	0.8389	p-Toluenesulfonic Acid Monohydrate	Antimalarial
Cl.Cl.O=C(O)COCCN1CCN(C(c2ccccc2)c2ccc(Cl)cc2)CC1	0.1801	0.8199	Cetirizine Dihydrochloride	Antihistaminic
CCOC(=O)[C@H](CCc1ccccc1)N[C@@H](C)C(=O)N1Cc2ccccc2C[C@H]1C(=O)O.Cl	0.1854	0.8146	Quinapril Hydrochloride	Ace inhibitor
Cl.Cl.Clc1cccc(C(c2ccc3nc[nH]c3c2)n2ccnc2)c1	0.1954	0.8046	Liarozole Dihydrochloride	Retinoic Acid (Ra) metabolism-blocking agent (Ramba)
Cl.N=C(N)N	0.2027	0.7973	Guanidine Hydrochloride	Protein denaturant

Structural analysis of the TACE protein

TACE consists of a single chain of 266 amino acids, comprising α-helices, β-sheets, and coils (Fig 5). According to VADAR statistical analysis, TACE's composition includes 30% α-helices, 27% β-sheets, 42% coils, and 22% turns. Moreover, the Ramachandran plot analysis indicated that 96.2% of all residues fall within favored regions, 100% are in allowed regions, and there are no outliers for the dihedral angles phi (φ) and psi (ψ).

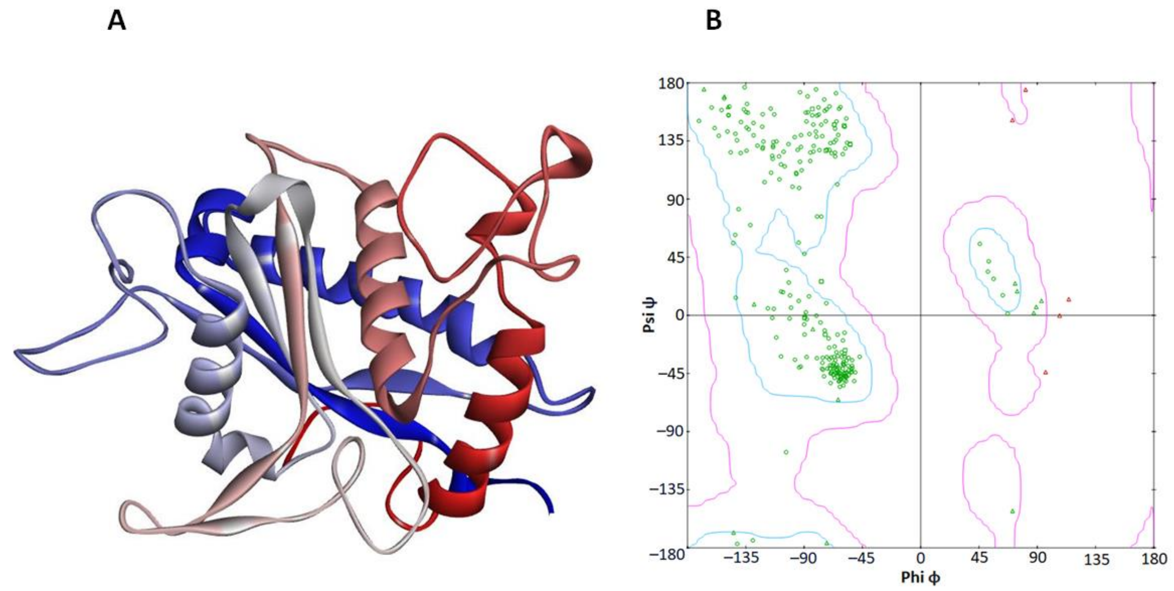

Fig 5. N-terminal to C-terminal 3D structure (A) of the TACE enzyme and the computed Ramachandran plot (B) showing 0 outliers.

The binding pocket analysis

The binding pocket is a structurally defined site on a protein, determined by its shape, location, and the assembly of amino acid residues that dictate its functionality [43]. The binding pocket residues of TACE were obtained using Discovery Studio's ligand interaction approach in which the 2OI0 and co-crystallized ligand was utilized to figure out the interacting amino acid residues and mentioned as Thr347, Leu348, Leu401, His405, Glu406, His409, Ala439, His415, Val434, Tyr436, and Pro437. Consequently, the verification of active pocket residues was done by comparing them with already published data [27]. Therefore, the binding sphere values were adjusted as X=44.4357, Y=-28.4975, Z=3.0389, and the radius value was fixed as 8.1601 concerning the binding pocket residues, to carry out the molecular docking of screened FDA drugs against the active region of target protein (Fig 6).

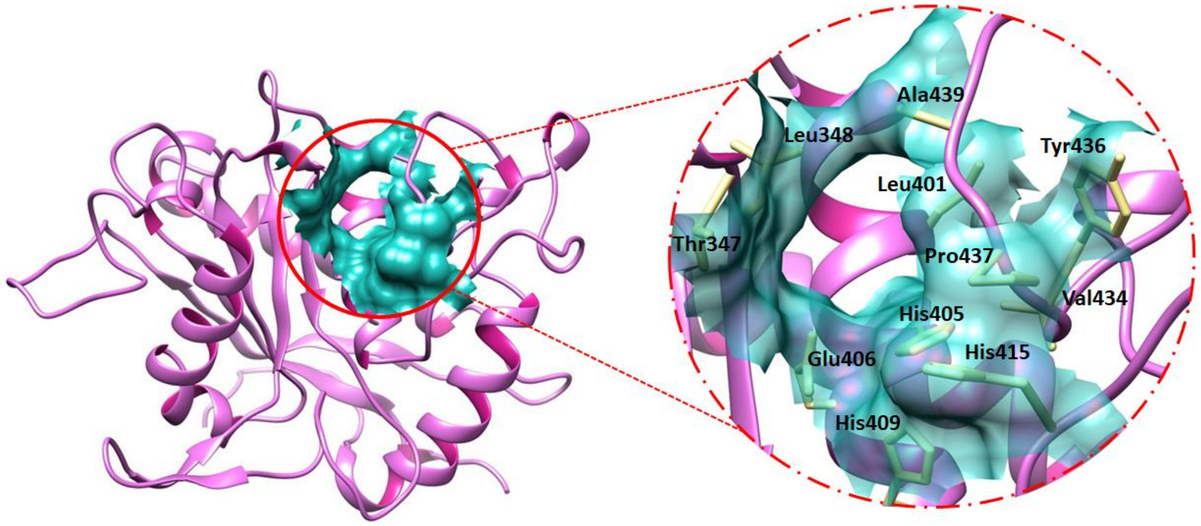

Fig 6. Figure manifests the real-time location of the active region of TACE on the left while the active pocket amino acids are depicted on the right side of the image.

Molecular docking analysis

The CDocker module of Discovery Studio was employed for molecular docking. CDocker energy represents the overall docking energy calculated from the 3D structural and physicochemical characteristics of the ligand and protein complex. On the other hand, CDocker

interaction energy specifically quantifies the energy associated with interactions, including hydrogen bonding, van der Waals forces, and electrostatic interactions [9]. From the subset of top screened compounds, 33 compounds including screened FDA drugs and a reference compound BMS-561392 were ranked based on their minimum docking energy in molecular docking studies (S1 Table).

Quinapril, Vorinostat, and Alvimopan demonstrated the lowest CDocker energy (-63.0703, -54.7973, -52.7959 kcal/mol respectively) among the compounds analyzed, suggesting they have the highest binding strength in molecular docking studies. They also exhibited notably low CDocker interaction energy marking it as a highly potent candidate against TACE. Although Vorinostat and Alvimopan had a slightly higher CDocker energy than Quinapril, they showed very favorable interaction energy (-60.0783, -65.4044 kcal/mol respectively), indicating robust interactions within the binding site. Marimastat, a known TACE inhibitor, recorded a CDocker energy of -49.1011 kcal/mol, reflecting its good binding affinity, though it was slightly higher than other top contenders. BMS-561392, the reference drug, had a CDocker energy of -46.8811 kcal/mol, which was relatively higher, yet it displayed the lowest interaction energy (-84.7487 kcal/mol) among all the compounds, suggesting exceptionally favorable interactions despite its overall binding affinity being lower.

Other compounds such as Bufexamac (-44.9771 kcal/mol), Loxoprofen (-44.2524 kcal/mol), and Thiamine (-41.7703 kcal/mol) also showed competitive CDocker energies, indicating their potential as effective inhibitors, although their interaction energies varied, with Loxoprofen having the most favorable among them. The significance of these findings extends to drug development, where compounds with lower CDocker energy values are generally preferred due to their higher likelihood of effective binding and stability. Quinapril, Vorinostat, and Alvimopan with their exceptionally low CDocker energy, emerge as a promising candidate for further investigation.

Similarly, Marimastat, BMS-561392, Loxoprofen, Bufexamac, and Thiamine with their relatively good binding and interaction profiles, could be strong candidates for therapeutic

applications. Despite its higher CDocker energy, BMS-561392's extremely low interaction energy may still render it a potent option against the target protein (Table 2).

Table 2. The CDocker and CDocker interactions score of the top 15 Docked compounds against TACE.

No	Compounds	CDocker energy (kcal/mol)	CDocker interaction energy (kcal/mol)
1	Quinapril	-63.0703	-79.1213
2	Vorinostat	-54.7973	-60.0783
3	Alvimopan	-52.7959	-65.4044
4	Marimastat	-49.1011	-57.4456
5	BMS-561392 (Ref)	-46.8811	-84.7487
6	Bufexamac	-44.9771	-51.3456
7	Loxoprofen	-44.2524	-58.5632
8	Thiamine	-41.7703	-45.0779
9	Adrafinil	-41.0990	-50.5074
10	Naproxen	-37.2369	-53.9294
11	Phenformin	-36.9916	-41.0075
12	Zinc_Acetate	-35.5984	-33.0282
13	Cetirizine	-34.6891	-54.0816
14	Liarozole	-32.0840	-45.1788
15	Detomidine	-31.7856	-34.8698

Understanding these energy values is crucial as they help in predicting the efficacy of the compounds in a biological setting. Compounds that bind more stably are less likely to dissociate from the target, enhancing their potential as effective drugs. The data provided here serves as a foundational step in the selection and optimization of lead compounds for further analysis.

Binding interaction analysis of screened Enamine compounds

The molecular docking results were further analyzed by examining the interactions between the compounds and the active region amino acids target protein, TACE, along with the corresponding binding distances in coordination with Zn2+. BMS-561392, the reference drug, interacted with Glu406 and Gly349, showing binding distances ranging from 1.97Å to 2.72Å. This interaction correlates with its low CDocker interaction energy, despite its relatively higher

CDocker energy. Quinapril, which had the lowest CDocker energy, formed interactions with Gly346, Leu348, and Gly349, with binding distances between 2.02Å and 2.68Å, supporting its strong binding interaction. Vorinostat, which also exhibited low CDocker energy, interacted with Gly349, Glu406, and Tyr436, with binding distances from 2.06Å to 2.64Å, highlighting its robust interaction profile. Alvimopan, another strong candidate, interacted with Gly349 and Leu348, with distances of 2.68Å and 2.39Å, respectively, which aligns with its favorable interaction energy. Marimastat, with its slightly higher CDocker energy, interacted closely with Gly349 and Glu406, with binding distances of 1.91Å and 2.23Å, reflecting its known inhibitory effects. Bufexamac, Loxoprofen, and Thiamine, which had competitive CDocker energies, interacted primarily with Gly349, His405, and Glu406, respectively, with binding distances around 2.00Å to 2.27Å, indicating their potential efficacy (Fig 7). These binding interactions provide insight into the molecular basis of the docking results, where compounds with lower CDocker energies and shorter binding distances generally exhibit more stable and effective interactions, highlighting their potential as therapeutic agents (Table 3).

The Zn2+ ion plays a pivotal role in the molecular docking and binding interactions within the active site of TACE. As a metalloproteinase, TACE's enzymatic activity and structural stability are heavily dependent on the coordination environment of the Zn2+ ion, which serves as a critical cofactor. The Zn2+ ion typically coordinates with key amino acid residues, such as Glu406, His405, and Gly349, forming a stable, catalytically active site that is crucial for ligand binding. In molecular docking studies, the presence of Zn2+ significantly influences the binding affinity and orientation of ligands. Compounds that can effectively interact with Zn2+ or its coordinating residues often exhibit more stable and potent binding conformation. Most of the ligands formed the interaction with the neighboring amino acids of Zn2+ thus showing the potency of the screened ligands to be further evaluated.

Table 3. The interacting amino acids and the bond lengths are manifested in this table of the top-screened FDA compounds.

**Drugs**	**Interacting amino acids**	**Binding distances**	**Common residues**

BMS-561392	Glu406<br>Gly349	2.03Å, 2.29Å<br>1.97Å, 2.72Å	Glu406, Gly349, Leu348
Quinapril	Gly346<br>Leu348<br>Gly349	2.02Å<br>2.04Å<br>2.68Å	
Vorinostat	Gly349<br>Glu406<br>Tyr436	2.64Å<br>2.27Å, 2.21Å<br>2.06Å	
Alvimopan	Gly349<br>Leu348	2.68Å<br>2.39Å	
Marimastat	Gly349<br>Glu406	1.91Å<br>2.23Å	
Bufexamac	Gly349	2.18Å	
Loxoprofen	His405	2.27Å	
Thiamine	Glu406	2.00Å	

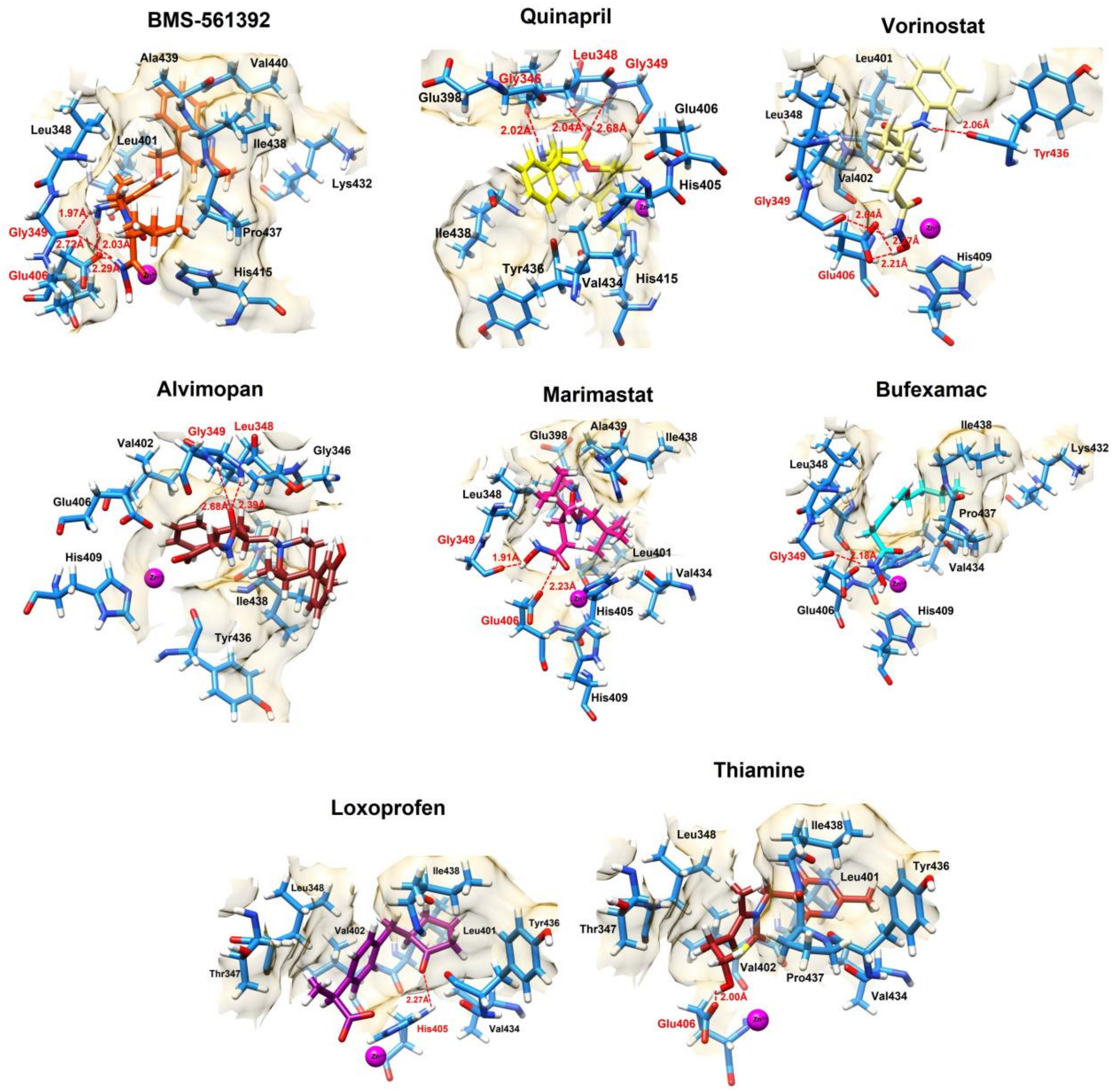

Fig 7. The 3D interactions of the docked ligands were depicted in this figure, confirming that the ligands bound in the active region of the target protein. The interactions, including hydrogen bonds and interacting amino acids, are depicted in red. The interacting Zn2+ ion crucial for TACE binding was colored magenta.

Molecular dynamics (MD) simulations

MD simulation reveals potential changes in binding modes, and the durability of interactions under physiological conditions, and identifies any transient states that contribute to their inhibitory activity. To assess the stability of the screened compounds against TACE, the docked complexes were subjected to 100ns MD simulations using GROMACS.

Root mean square deviation

The molecular dynamics trajectories were analyzed by computing the Root Mean Square Deviation (RMSD) to evaluate ligand fluctuations within the active site of the TACE protein. BMS-561392, Vorinostat, and Bufexamac exhibited similar behavior in terms of their RMSD values, showing the lowest RMSD and highly stable profiles throughout the simulation, suggesting a strong correlation with their favorable docking scores and interaction energies. This stability indicates that these compounds maintain consistent binding within the active site, reinforcing their potential as potent inhibitors. Thiamine and Quinapril, while also displaying stable RMSD profiles, had relatively higher RMSD values compared to Vorinostat and BMS-561392. Marimastat, despite its slightly higher CDocker energy, exhibited notable transitions in its RMSD profile. Initially, the RMSD increased, followed by a decrease at around 28ns, and then a re-increase, with a significant drop to 0.56Å at 85ns, which remained stable until the end of the simulation. This fluctuation might reflect Marimastat's binding dynamics, where the compound adjusts within the active site before stabilizing, potentially indicating a flexible binding mode that could influence its inhibitory efficacy. Alvimopan's RMSD profile revealed fluctuations from start to finish, with values oscillating between 0.3Å and 0.5Å, and a peak of 0.72Å at 8ns. This variability might be attributed to the compound's interactions within the binding site, which, while stable, involve slight positional shifts that are less stable than those seen in compounds like Vorinostat or BMS-561392. Loxoprofen, on the other hand, showed a relatively stable RMSD compared to Alvimopan and Marimastat, with only one significant peak between 39ns and 60ns before re-stabilizing. This suggests that while Loxoprofen's binding is generally stable, it may undergo transient conformational changes before achieving equilibrium (Fig 8).

The observed RMSD behaviors, when compared with the docking scores, suggest that compounds with lower RMSD values tend to have more stable and consistent binding, aligning with their lower CDocker energies. The stable RMSD values of Vorinostat and bufexamac revealed a strong interaction while fluctuations in RMSD for Marimastat and Alvimopan could indicate a more dynamic interaction within the binding site. These results imply that while stable RMSD profiles correlate with strong binding affinity, the flexibility seen in some

compounds may reflect the potential for adaptive binding mechanisms that could be further explored for therapeutic applications.

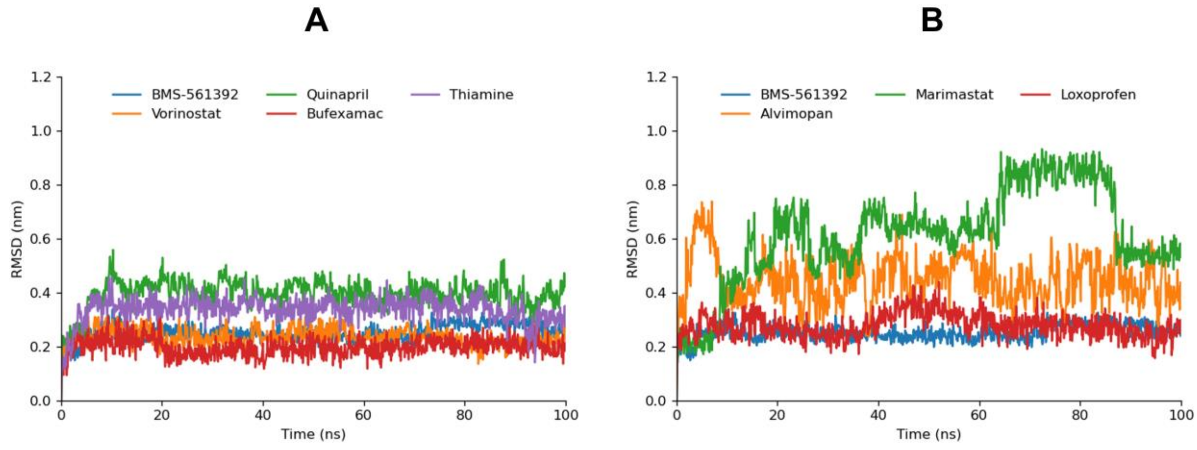

Fig 8. The graphs illustrate the RMSD plot of the ligands. The simulated ligands were colored differently for comparison. The less fluctuating bar graphs are depicted in graph Figure A and compounds with moderate to high fluctuations are manifested in Figure B.

In addition to analyzing the ligand-protein RMSD, backbone-to-backbone RMSD calculations were also conducted to assess whether the proteins reached equilibrium during the simulation (Fig 9). The resulting bar graphs revealed a consistent and stable behavior across the board, indicating that the overall protein structures maintained their integrity and did not undergo significant conformational changes. This stability in the backbone RMSD suggests that the proteins were well equilibrated throughout the simulation, providing a reliable framework for analyzing the ligand interactions.

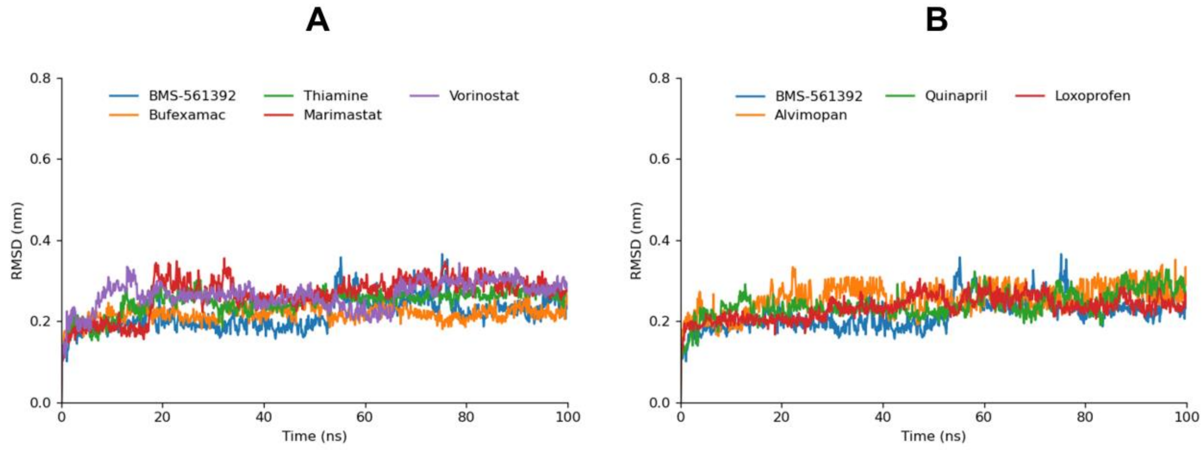

Fig 9. The graphs illustrate the RMSD plots of the protein backbone. Graph A manifests the RMSD backbone of Thiamine, Vorinostat, Bufexamac, and Marimastat compared to BMS-561392. Graph B exhibits the RMSD backbone of Quinapril, Alvimopan, and Loxoprofen compared to BMS-561392.

Hydrogen bond plot analysis

The hydrogen bond plot analysis distinguishes between two types of hydrogen bonds: actual hydrogen bonds, which were identified during the 100ns molecular dynamics (MD) trajectory, and potential hydrogen bonds, where the atoms of the ligands and receptor are close enough (within 0.35 nm) to possibly form hydrogen bonds in future trajectories (Figure 10).

The hydrogen bond plot analysis revealed significant insights into the binding dynamics of various compounds with the target protein, TACE. BMS-561392, the reference compound, consistently formed two actual and two potential hydrogen bonds, underscoring its strong and stable interaction with the target. Vorinostat also demonstrated robust binding throughout the 100 ns MD trajectory, forming three actual hydrogen bonds and one potential hydrogen bond, along with numerous additional peaks for potential hydrogen bonds, indicating its strong candidacy as a TACE inhibitor. Bufexamac showed stable interactions with two actual and one potential hydrogen bond, the peaks of 4th and 5th potential hydrogen bonds can also be seen. Marimastat also had two actual hydrogen bonds in the initial trajectory while after 80ns the number of actual hydrogen bonds were reduced but the number of potential hydrogen bonds remain consistent.

In comparison, Quinapril, Thiamine and Loxoprofen maintained one actual hydrogen bond, with the second actual and potential hydrogen bonds alternating, indicating intermittent interactions. Alvimopan on the initial trajectory manifested low interactions but after 55ns the number of interactions increased followed by the decrease at the end, corelating the RMSD. Quinapril, Thiamine and Loxoprofen showed weaker interactions overall, with Alvimopan notably increasing its potential hydrogen bonds in the second half of the simulation, suggesting a delayed or less binding effect. These findings align with the RMSD results, providing a comprehensive understanding of the binding dynamics and potential efficacy of these

compounds. Further studies are necessary to validate these observations and explore their therapeutic potential as TACE inhibitors in clinical settings.

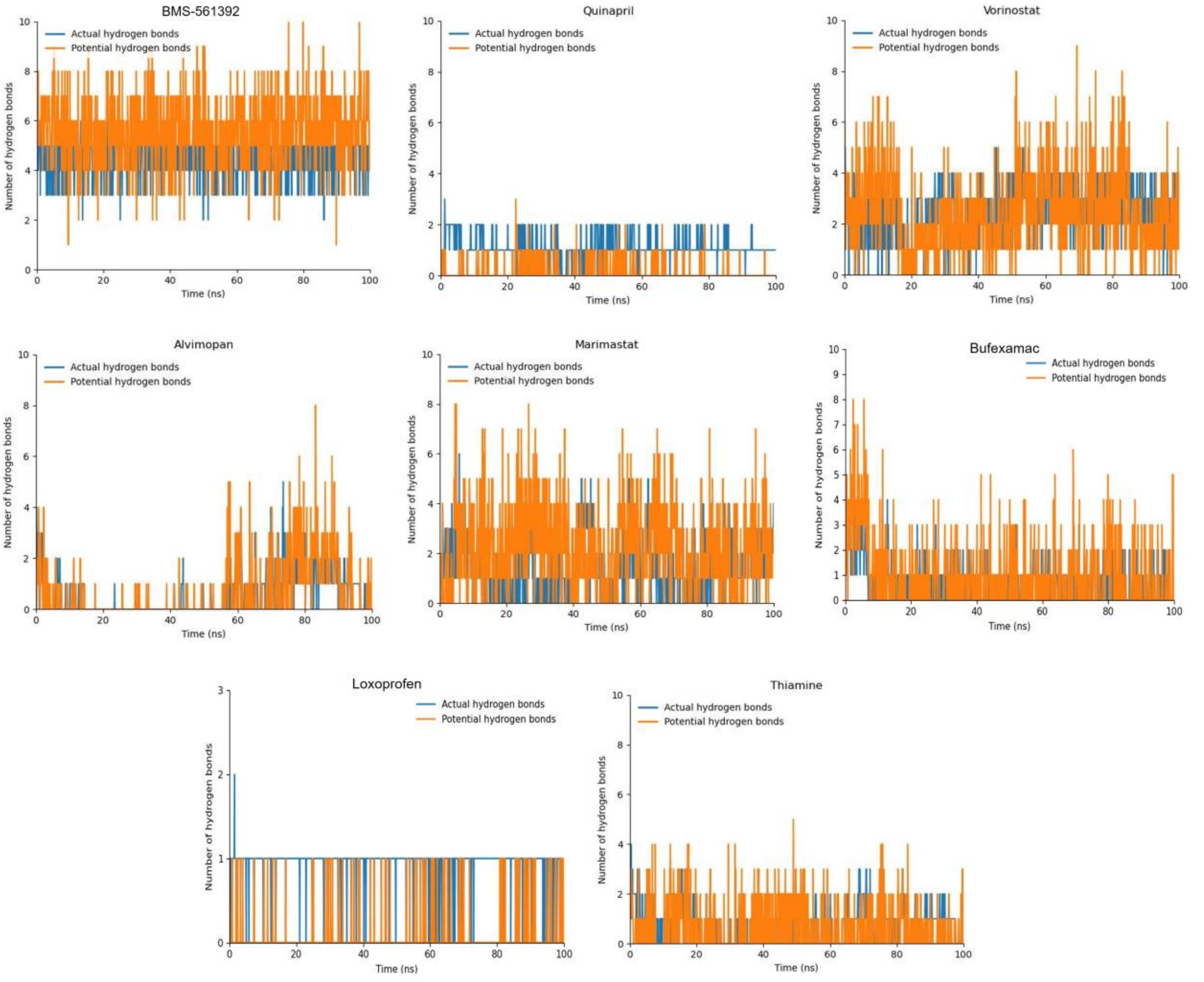

Fig 10. The graphs show the hydrogen bond plots of the top 10 docked compounds against TACE. The blue peaks are the actual hydrogen bonds while the orange peaks are potential hydrogen bonds.

Interaction energy analysis

In addition to hydrogen bond visualization and RMSD analysis, the interaction energies of the top 10 docked compounds were calculated against TACE during a 100ns MD simulation to evaluate the interaction energy scores of the docked complexes. The interaction energy consists of two components: electrostatic (Coulombic) interaction energy and Lennard-Jones interaction energy, with their sum representing the total interaction energy.

BMS-561392 as a reference drug exhibited the lowest total interaction energy (-103.1152 kcal/mol). Among the analyzed compounds, Vorinostat (-51.5201 kcal/mol) indicated the

strongest binding affinity, followed by Thiamine (-49.0065 kcal/mol). Quinapril, which had the lowest CDocker energy score, with a total energy of -42.7971 kcal/mol, also showed significant binding strength. In comparison, Loxoprofen had the highest total interaction energy (-0.6898 kcal/mol), suggesting weaker binding (Table 4). These interaction energy results correlate with RMSD and hydrogen bond analyses, which provide additional insights into the binding stability and specificity of these compounds. Vorinostat's strong binding affinity and comprehensive interaction profile, as reflected in both its low total interaction energy and favorable RMSD and hydrogen bond metrics, underscore its potential as a highly potent therapeutic agent. Bufexamac, while also displaying low interaction energies, may have slightly less potency compared to Vorinostat due to their fewer binding interactions as indicated by RMSD and hydrogen bond analyses. Overall, this integrated approach combining interaction energy data with structural and interaction analyses provides a robust framework for evaluating the efficacy of these compounds in drug development.

The sum of Coulombic and Lennard-Jones interaction energy (Total energy) of the screened FDA drugs was graphically depicted in Figure 11 in comparison with BMS-561392.

Table 4. The Electrostatic, Lennard-Jones contributions and the total energies are shown.

**No**	**Compound**	**Interaction energy (Kcal/mol)**		
		**Coul-SR**	**LJ-SR**	**Total energy**
	BMS-561392(Ref)	-51.1147	-52.0005	-103.1152
1	Vorinostat	-16.6317	-34.8884	-51.5201
2	Thiamine	-24.2450	-24.7615	-49.0065
3	Quinapril	-13.1931	-29.6040	-42.7971
4	Marimastat	-13.7749	-28.0409	-41.8158
5	Bufexamac	-9.33100	-24.9137	-34.2447
6	Alvimopan	-11.8919	-18.6340	-30.5259
7	Loxoprofen	30.3566	-31.0464	-0.6898

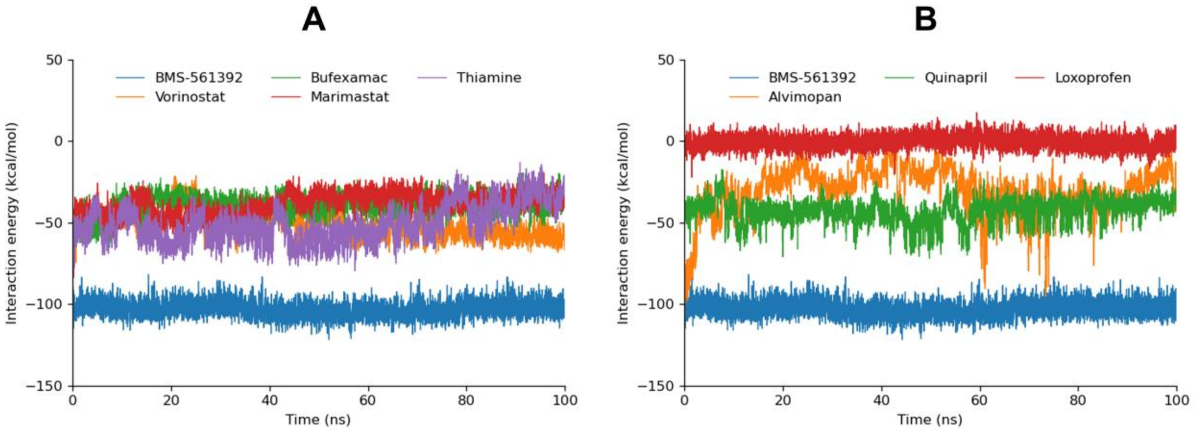

Fig 11. The computed total interaction energies of Enamine compounds, derived by summing the Lennard-Jones and Coulombic contributions, are presented here in comparison with BMS-561392.

gmx_MMPBSA Binding free energy calculation

The entire trajectories obtained during 100ns MD simulations was divided into five groups, therefore was subjected to calculate the binding free energy of docked complexes. The gmx_MMPBSA tool used with GROMACS was employed and the MM/PBSA method was used to calculate the binding energy with default parameters (Figure 12 (A, B)).

The average -ΔG values along with their Standard Deviation (SD) for screened compounds evaluated for their binding affinity to a target protein in comparison with the reference compound. Notably, Loxoprofen revealed very low binding affinity therefore removed from the data set. BMS-561392 exhibited the most favorable binding energy with ΔG of -40.72 kcal/mol, indicating strong binding affinity. This is complemented by a low average SD value of 3.54, suggesting consistency in the calculated energy values across simulations. Vorinostat also demonstrated significant binding affinity with ΔG -25.80 kcal/mol with a SD value of 3.67, indicating robustness in its binding interactions. Bufexamac showed ΔG values of -18.37 kcal/mol with moderate SD (3.42), suggesting stable but less favorable binding compared to BMS-561392 and Vorinostat. Furthermore, Marimastat and Quinapril displayed ΔG values of -17.41 kcal/mol and -15.04 kcal/mol with relatively lower SD values, indicating consistent but weaker binding affinities compared to BMS-561392 and Vorinostat (Table 5).

The ΔG values correlate with the interaction energies observed in the hydrogen bond plot analysis. Compounds with lower -ΔG, such as BMS-561392 and Vorinostat, generally exhibited more stable hydrogen bond interactions with the target protein over the 100 ns MD simulation. Conversely, compounds like Thiamine and Alvimopan, which showed higher -ΔG values of -8.97 kcal/mol and -13.34 kcal/mol respectively, also displayed less favorable and less stable hydrogen bond patterns. Overall, these findings highlight Vorinostat as a promising candidate for further investigation as a TACE inhibitor, supported by its strong -ΔG values and stable interaction profiles followed by Bufexamac. Further experimental validation is crucial to confirm these computational predictions and evaluate their potential therapeutic efficacy.

Table 5. The binding affinity of the top 10 simulated compounds in comparison with BMS-561392.

**Compound**	**1ns-20ns**		**20ns-40ns**		**40ns-60ns**		**60ns-80ns**		**80ns-100ns**		**Average**	
	**ΔG**	**SD**	**ΔG**	**SD**	**ΔG**	**SD**	**ΔG**	**SD**	**ΔG**	**SD**	**ΔG**	**SD**
BMS-561392	-39.01	5.09	-41.43	2.96	-41.90	4.58	-39.77	2.36	-41.51	2.71	-40.72	3.54
Vorinostat	-20.45	5.68	-21.41	3.77	-28.39	3.12	-29.40	2.03	-29.33	3.74	-25.80	3.67
Bufexamac	-18.70	4.91	-20.09	3.55	-19.69	2.61	-17.01	3.41	-16.39	2.60	-18.37	3.42
Marimastat	-15.04	3.73	-16.86	4.18	-16.51	2.61	-16.28	3.29	-22.38	4.29	-17.41	3.62
Quinapril	-16.56	3.23	-15.25	5.10	-15.65	3.98	-15.00	3.56	-12.72	3.01	-15.04	3.77
Alvimopan	-15.17	3.80	-13.33	2.50	-11.69	2.64	-14.06	5.04	-12.49	3.70	-13.34	3.53
Thiamine	-12.21	3.35	-8.41	5.03	-8.88	5.27	-8.370	4.94	-7.00	3.79	-8.97	4.48

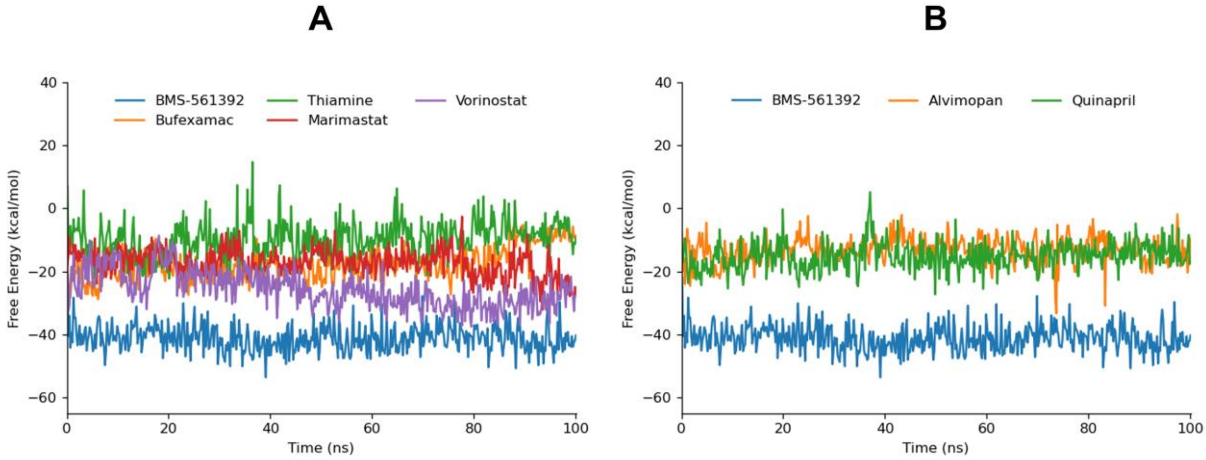

Fig 12. The graphical depiction of gmx_MMPBSA free energy calculation of top 6 compounds in comparison with BMS-561392.

Structural evaluation and similarity comparison

To evaluate the structural similarities among the selected compounds for experimental assessment, we utilized RDKit's Tanimoto similarity measure. The analysis indicated that there was no significant structural resemblance between the selected compounds and BMS-561392, as shown by the Tanimoto similarity (Table 6). While there is no strict threshold for defining similarity, a Tanimoto similarity score of 0.8 or higher generally suggests similarity, ranging from 0 (no similarity) to 1 (complete similarity). Although the selected compounds exhibited limited overall similarity to BMS-561392, they may still share specific common structural motifs. To identify these common substructures, we employed the Maximum Common Substructure (MCS) algorithm in RDKit, which utilizes SMARTS (SMILES Arbitrary Target Specification). No recurring structural motifs were identified, aside from the benzene ring highlighted in green (Figure 13). This observation suggests that factors beyond structural motifs, such as the spatial orientation of specific conformations, could potentially influence the inhibitory activity against TACE. Additionally, we utilized RDKit fingerprints to generate similarity maps to determine if the selected compounds contain structural motifs similar to those in BMS-561392 (Figure 13). The similarity maps indicated the presence of BMS-561392 structural motifs within the chemical structures of the selected compounds. These insights from the MCS and similarity mapping provide valuable guidance for further optimizing the selected compounds.

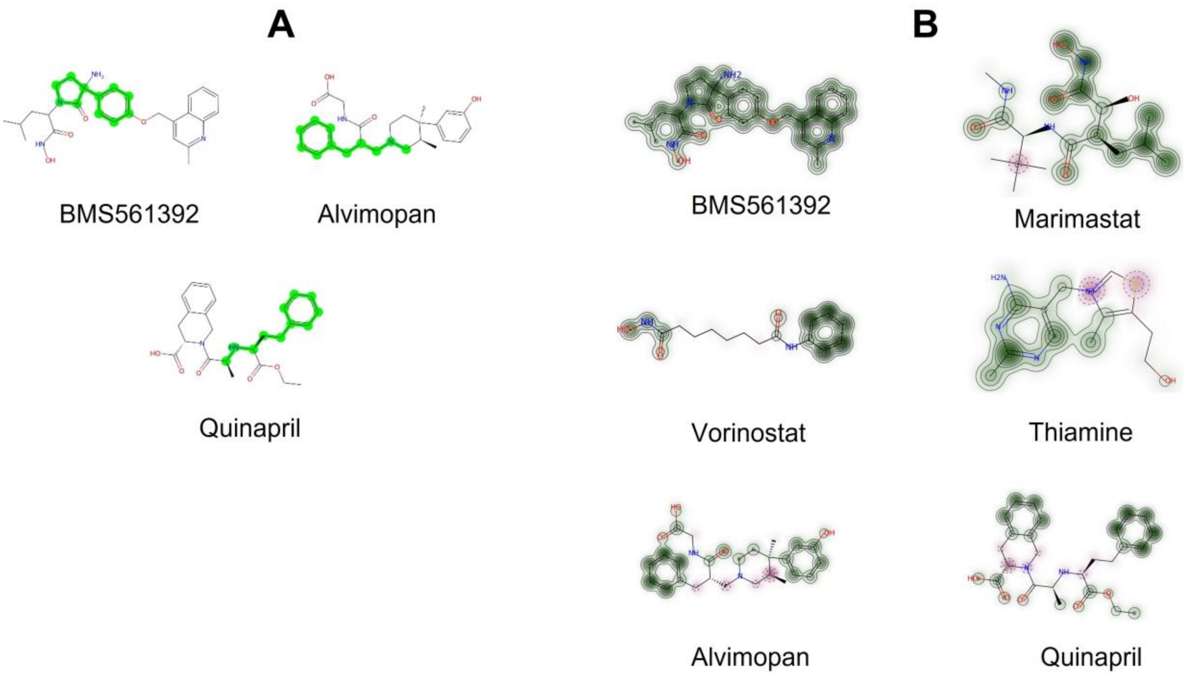

Figure 13. Visual depiction of the common structural motif identified through Maximum Common Substructure (MCS) analysis using SMARTS (A), and graphical representation illustrating the shared common structural motif through similarity maps (B).

Table 6. Tanimoto similarity comparison of selected compounds.

Names	BMS-561392	Marimastat	Vorinostat	Bufexamac	Thiamine	Alvimopan	Quinapril
BMS-561392	-	0.313	0.123	0.205	0.280	0.348	0.444
Marimastat	0.313	-	0.172	0.123	0.181	0.268	0.283
Vorinostat	0.123	0.172	-	0.150	0.096	0.124	0.102
Bufexamac	0.205	0.123	0.150	-	0.127	0.210	0.169
Thiamine	0.280	0.181	0.096	0.127	-	0.187	0.235
Alvimopan	0.348	0.268	0.124	0.210	0.187	-	0.311
Quinapril	0.444	0.283	0.102	0.169	0.235	0.311	-

The computational studies result were well presented but the discussion needs to be improved by supporting the findings with proper referencing of another source to support their discussion.

Experimental validation

To validate the computational findings, the TACE inhibitory activity of six compounds was assessed by measuring the release level of TNF-α at a concentration of 10 μM in RAW 263.7 macrophage cells. BMS-561392, used as the reference compound, showed the lowest TNF-α release, indicating the strongest inhibition of TACE. Marimastat, another known TACE inhibitor that was also highly predicted by the GraphConvMol algorithm in the present study,

demonstrated significant suppression of TACE. Notably, Vorinostat exhibited potent inhibitory activity on TACE (Fig 14). Vorinostat is an anticancer medication used to treat cutaneous T-cell lymphoma by inhibiting histone deacetylase (HDAC) [44-46]. Prior to this study, there were no reports of Vorinostat affecting TACE activity. In contrast, Thiamine showed the least effect, suggesting weaker inhibition. Other compounds, including Bufexamac and Quinapril displayed moderate to high enzymatic activities, reflecting a range of inhibitory potential.

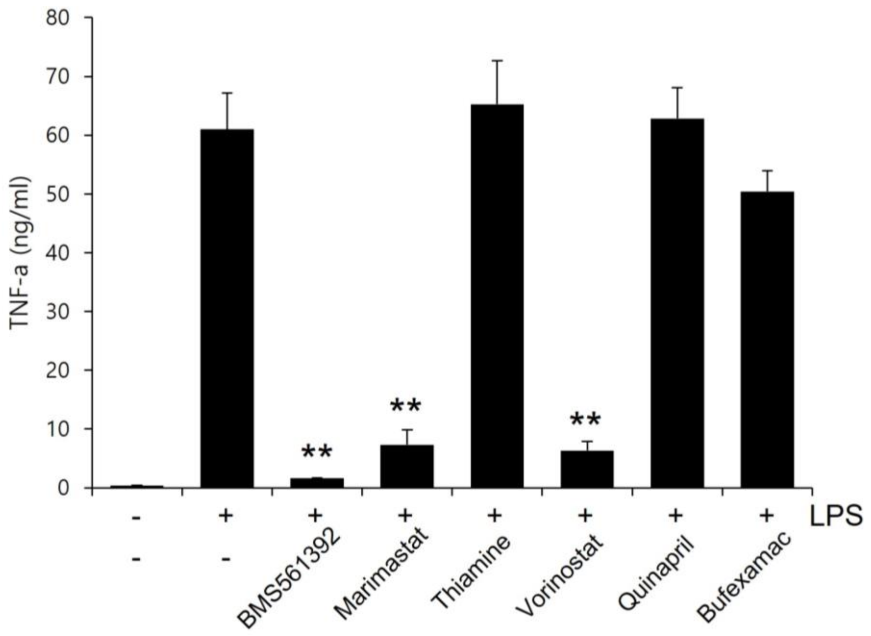

Fig 14. TACE inhibitory activity of selected compounds in comparison to BMS-561392.

The enzymatic activity results align well with the gmx_MMPBSA and MD simulation outcomes. BMS-561392, which showed the lowest enzymatic activity, also had the most favorable binding energies and stability in MD simulations, supporting its strong inhibitory effect on TACE. Vorinostat, which demonstrated significant inhibition in enzymatic assays, was predicted to have strong binding affinity and stability in the gmx_MMPBSA and MD simulations, reinforcing its potential as a TACE inhibitor. Marimastat's moderate enzymatic activity corresponds with its consistent performance in computational analyses, validating its known efficacy. Conversely, Thiamine's low enzymatic activity, indicating weak inhibition, is consistent with its lower binding affinity in the gmx_MMPBSA. Other compounds like Quinapril and Bufexamac showed moderate binding affinities and stabilities in MD simulations,

which correlate with their enzymatic activity results, highlighting their varied inhibitory potentials. This comprehensive analysis demonstrates the reliability of integrating computational and experimental approaches.

Conclusion

In conclusion, this study demonstrates the powerful application of deep learning models in the drug discovery process, particularly in identifying novel inhibitors for specific targets such as TACE. By utilizing advanced cheminformatics tools and a robust predictive framework, we successfully screened a library of FDA-approved drugs and identified several promising TACE inhibitors, including Vorinostat, Quinapril, Alvimopan, and Marimastat. Vorinostat, previously known as an anticancer medication that works by inhibiting histone deacetylase, has not been reported to inhibit TACE prior to our findings. Our computational predictions, supported by molecular docking experiments, confirmed Vorinostat's strong binding affinity and inhibitory activity against TACE, suggesting its potential for repurposing in the treatment of inflammatory diseases like rheumatoid arthritis. Similarly, Quinapril demonstrated the lowest docking energy, indicative of its strong potential to bind and inhibit TACE effectively. Alvimopan and Marimastat also exhibited favorable binding properties, with molecular docking and dynamic simulation studies revealing stable interactions with critical residues in the TACE active site.

These findings underscore the efficiency and effectiveness of AI-driven approaches in accelerating drug repurposing and discovery efforts. The identification of potential TACE inhibitor, such as Vorinostat, opens new avenues for therapeutic intervention and highlights the versatility of this compound beyond its original indications. This drugs demonstrated unique interaction profiles with the TACE active site suggesting a diverse mechanism of inhibition that warrants further experimental validation and optimization.

Declarations:

Author Contributions: MY and JYP were involved in the experimental operation and data analysis. ETH, WSP, and JHH were involved in data curation and the methodology. WC was

involved in the conceptualization, and writing of the manuscript. MY and WC confirmed the authenticity of all the raw data. MH, AK, and WC were involved in reviewing and editing the manuscript. All authors have read and approved the final manuscript.

Funding: This work was supported by a Research Grant from the Institute of Medical Sciences, Kangwon National University 2024, a Korea Basic Science Institute (National Research Facilities and Equipment Center) grant funded by the Ministry of Education (grant no. 2022R1A6C101A739), and NIH grant numbers R01GM127701 and R01HG012117.

Institutional Review Board Statement: Not applicable.

Conflicts of Interest: The authors declare that they have no competing interests.

Acknowledgment: WC acknowledges the Research Grant from the Institute of Medical Sciences, Kangwon National University 2024 and a Korea Basic Science Institute (National Research Facilities and Equipment Center) grant funded by the Ministry of Education (grant no. 2022R1A6C101A739). A.K. acknowledges financial support from NIH Grants R01 GM127701 and R01HG012117. M.H. acknowledges The Ohio State University for the "President's Postdoctoral Scholars Program (PPSP)" fellowship for financial support.

Data Availability Statement: The data that support the findings of this study are available from the corresponding author upon reasonable request.

References

1. Illahi MI, Amjad S, Alam SM, Ahmed ST, Fatima M, Shahid MAJC. Serum tumor necrosis factor-alpha as a competent biomarker for evaluation of disease activity in early rheumatoid arthritis. Cureus. 2021;13(5).
2. Farrugia M, Baron BJJoc, research t. The role of TNF-α in rheumatoid arthritis: a focus on regulatory T cells. J Clin Transl Res. 2016;2(3):84.
3. Calzascia T, Pellegrini M, Hall H, Sabbagh L, Ono N, Elford AR, et al. TNF-α is critical for antitumor but not antiviral T cell immunity in mice. The Journal of Clinical Investigation. J Clin Invest. 2007;117(12):3833-45. doi: 10.1172/JCI32567.
4. Mercogliano MF, Bruni S, Mauro F, Elizalde PV, Schillaci R. Harnessing Tumor Necrosis Factor Alpha to Achieve Effective Cancer Immunotherapy. Cancers. 2021;13(3). Epub 2021/02/06. doi: 10.3390/cancers13030564. PubMed PMID: 33540543; PubMed Central PMCID: PMCPMC7985780.
5. Sethi JK, Hotamisligil GS. Metabolic Messengers: tumour necrosis factor. Nature Metabolism. 2021;3(10):1302-12. doi: 10.1038/s42255-021-00470-z.
6. Li W, Liu Q, Shi J, Xu X, Xu J. The role of TNF-α in the fate regulation and functional reprogramming of mesenchymal stem cells in an inflammatory microenvironment. Front Immunol. 2023;14. doi: 10.3389/fimmu.2023.1074863.
7. Moelants EA, Mortier A, Van Damme J, Proost PJI, biology c. Regulation of TNF-α with a focus on rheumatoid arthritis. Immunol Cell Biol. 2013;91(6):393-401.
8. Montgomery SL, Bowers WJJJonp. Tumor necrosis factor-alpha and the roles it plays in homeostatic and degenerative processes within the central nervous system. J Neuroimmune Pharmacol. 2012;7(1):42-59.
9. Murumkar PR, Ghuge RB, Chauhan M, Barot RR, Sorathiya S, Choudhary KM, et al. Recent developments and strategies for the discovery of TACE inhibitors. Expert Opin Drug Discov. 2020;15(7):779-801.
10. Murumkar PR, DasGupta S, Chandani SR, Giridhar R, Yadav MRJEootp. Novel TACE inhibitors in drug discovery: a review of patented compounds. Expert Opin Ther Pat. 2010;20(1):31-57.
11. Srinivas AN, Suresh D, Vishwanath PM, Satish S, Santhekadur PK, Koka S, et al. TACE inhibition: a promising therapeutic intervention against AATF-mediated steatohepatitis to hepatocarcinogenesis. Mol Oncol. 2024.
12. O'reilly S, Cant R, Ciechomska M, Van Laar JMJC, Immunology T. Interleukin-6: a new therapeutic target in systemic sclerosis? Clin Transl Immunology. 2013;2(4):e4.
13. Ara T, DeClerck YA. Interleukin-6 in bone metastasis and cancer progression. European Journal of Cancer. Eur J Cancer. 2010;46(7):1223-31. doi: https://doi.org/10.1016/j.ejca.2010.02.026.
14. Zielinski MR, Gibbons AJ. Neuroinflammation, sleep, and autoimmune disease. In: Kushida CA, editor. Encyclopedia of Sleep and Circadian Rhythms (Second Edition). Oxford: Academic Press; 2023. p. 258-71.
15. Li C, Gu H, Yu M, Yang P, Zhang M, Ba H, et al. Inhibition of transmembrane TNF-α shedding by a specific antibody protects against septic shock. Cell Death Dis. 2019;10(8):586. doi: 10.1038/s41419-019-1808-6.
16. Wang SE, Xiang B, Guix M, Olivares MG, Parker J, Chung CH, et al. Transforming growth factor beta engages TACE and ErbB3 to activate phosphatidylinositol-3 kinase/Akt in ErbB2-overexpressing breast cancer and desensitizes cells to trastuzumab. Mol Cell Biol. 2008;28(18):5605-20. Epub 2008/07/16. doi: 10.1128/mcb.00787-08. PubMed PMID: 18625725; PubMed Central PMCID: PMCPMC2546920.
17. Tsakadze N, Sithu S, Sen U, English W, Murphy G, D'Souza S. Tumor Necrosis Factor-α-converting

Enzyme (TACE/ADAM-17) Mediates the Ectodomain Cleavage of Intercellular Adhesion Molecule-1 (ICAM-1). J Biol Chem. 2006;281:3157-64. doi: 10.1074/jbc.M510797200.

18. Sun Q, Hampel H, Blennow K, Lista S, Levey A, Tang B, et al. Increased plasma TACE activity in subjects with mild cognitive impairment and patients with Alzheimer's disease. J Alzheimers Dis. 2014;41(3):877-86.
19. McCoy MK, Tansey MG. TNF signaling inhibition in the CNS: implications for normal brain function and neurodegenerative disease. J Neuroinflammation. 2008;5(1):45. doi: 10.1186/1742-2094-5-45.
20. Bahia MS, Silakari OJCb, design d. Tumor necrosis factor alpha converting enzyme: an encouraging target for various inflammatory disorders. Chem Biol Drug Des. 2010;75(5):415-43.
21. G Li N, H Shi Z, P Tang Y, A Duan JJCmc. Discovery of selective small molecular TACE inhibitors for the treatment of rheumatoid arthritis. Curr Med Chem. 2012;19(18):2924-56.
22. Murumkar PR, Giridhar R, Yadav MRJEOoDD. Novel methods and strategies in the discovery of TACE inhibitors. Expert Opin Drug Discov. 2013;8(2):157-81.
23. Barbosa MLdC, Fumian MM, Miranda ALPd, Barreiro EJ, Lima LMJBJoPS. Therapeutic approaches for tumor necrosis factor inhibition. Braz J Pharmaceutical Sci. 2011;47:427-46.
24. Pettersen EF, Goddard TD, Huang CC, Couch GS, Greenblatt DM, Meng EC, et al. UCSF Chimera—a visualization system for exploratory research and analysis. J Comput Chem. 2004;25(13):1605-12.
25. Jejurikar BL, Rohane SH. Drug designing in discovery studio. 2021.
26. Yasir M, Park J, Han E-T, Park WS, Han J-H, Kwon Y-S, et al. Vismodegib Identified as a Novel COX-2 Inhibitor via Deep-Learning-Based Drug Repositioning and Molecular Docking Analysis. ACS Omega. 2023;8(37):34160-70. doi: 10.1021/acsomega.3c05425.
27. Govinda Rao B, Bandarage UK, Wang T, Come JH, Perola E, Wei Y, et al. Novel thiol-based TACE inhibitors: rational design, synthesis, and SAR of thiol-containing aryl sulfonamides. Bioorg Med Chem Lett. 2007;17(8):2250-3. Epub 2007/02/10. doi: 10.1016/j.bmcl.2007.01.064. PubMed PMID: 17289381.
28. Yasir M, Park J, Han E-T, Park WS, Han J-H, Kwon Y-S, et al. Investigation of Flavonoid Scaffolds as DAX1 Inhibitors against Ewing Sarcoma through Pharmacoinformatic and Dynamic Simulation Studies. Int J Mol Sci. 2023;24(11):9332. PubMed PMID: doi:10.3390/ijms24119332.
29. Yasir M, Park J, Han E-T, Park WS, Han J-H, Chun WJM. Drug Repositioning via Graph Neural Networks: Identifying Novel JAK2 Inhibitors from FDA-Approved Drugs through Molecular Docking and Biological Validation. Molecules. 2024;29(6):1363.
30. Yasir M, Park J, Han ET, Park WS, Han JH, Kwon YS, et al. Machine Learning-Based Drug Repositioning of Novel Janus Kinase 2 Inhibitors Utilizing Molecular Docking and Molecular Dynamic Simulation. J Chem Inf Model. 2023;63(21):6487-500. Epub 2023/10/31. doi: 10.1021/acs.jcim.3c01090. PubMed PMID: 37906702.
31. Jo S, Kim T, Iyer VG, Im WJJocc. CHARMM-GUI: a web-based graphical user interface for CHARMM. J Comput Chem. 2008;29(11):1859-65.
32. Huang J, Rauscher S, Nawrocki G, Ran T, Feig M, de Groot BL, et al. CHARMM36: An improved force field for folded and intrinsically disordered proteins. Nat Methods 2017;112(3):175a-6a.
33. Mark P, Nilsson LJTJoPCA. Structure and dynamics of the TIP3P, SPC, and SPC/E water models at 298 K. J Physic Chem. 2001;105(43):9954-60.
34. Berendsen HJ, van der Spoel D, van Drunen RJCpc. GROMACS: A message-passing parallel molecular dynamics implementation. PloS Comput Biol. 1995;91(1-3):43-56.

35. Wang E, Sun H, Wang J, Wang Z, Liu H, Zhang JZ, et al. End-point binding free energy calculation with MM/PBSA and MM/GBSA: strategies and applications in drug design. Chem Rev. 2019;119(16):9478-508.
36. Mysinger MM, Carchia M, Irwin JJ, Shoichet BKJJomc. Directory of useful decoys, enhanced (DUD-E): better ligands and decoys for better benchmarking. J Med Chem. 2012;55(14):6582-94.
37. Zhang Y, Vass M, Shi D, Abualrous E, Chambers JM, Chopra N, et al. Benchmarking refined and unrefined AlphaFold2 structures for hit discovery. J Chem Inf Model. 2023;63(6):1656-67.
38. Sabe VT, Ntombela T, Jhamba LA, Maguire GE, Govender T, Naicker T, et al. Current trends in computer aided drug design and a highlight of drugs discovered via computational techniques: A review. Eur J Med Chem. 2021;224:113705.
39. Minnich AJ, McLoughlin K, Tse M, Deng J, Weber A, Murad N, et al. AMPL: a data-driven modeling pipeline for drug discovery. J Chem Info Model. 2020;60(4):1955-68.
40. Ramsundar B, Eastman P, Walters P, Pande V. Deep learning for the life sciences: applying deep learning to genomics, microscopy, drug discovery, and more: " O'Reilly Media, Inc."; 2019.
41. Grebner C, Matter H, Kofink D, Wenzel J, Schmidt F, Hessler GJC. Application of deep neural network models in drug discovery programs. ChemMedChem. 2021;16(24):3772-86.
42. Grebner C, Matter H, Hessler GJAIiDD. Artificial intelligence in compound design. Methods Mol Biol. 2022:349-82.
43. Yasir M, Park J, Chun WJAo. Discovery of Novel Aldose Reductase Inhibitors via the Integration of Ligand-Based and Structure-Based Virtual Screening with Experimental Validation. ACS Omega. 2024;9(18):20338-49.
44. Sato AJO, therapy. Vorinostat approved in Japan for treatment of cutaneous T-cell lymphomas: status and prospects. Onco Targets Ther. 2012:67-76.
45. Tiffon C, Adams J, Van Der Fits L, Wen S, Townsend P, Ganesan A, et al. The histone deacetylase inhibitors vorinostat and romidepsin downmodulate IL-10 expression in cutaneous T-cell lymphoma cells. Br J Pharmacol. 2011;162(7):1590-602.
46. Rangwala S, Duvic M, Zhang CJB, Targets LC, Therapy. Trends in the treatment of cutaneous T-cell lymphoma–critical evaluation and perspectives on vorinostat. Blood Lymphatic Cancer. 2012:17-27.

Click here to access/download
Supporting Information
Yasir_Supplementary_Data.docx
