# Supplementary material for: Discovery of novel TACE inhibitors using graph convolutional network, molecular docking, molecular dynamics simulation, and Biological evaluation
Source: PLoS One. 2024 Dec 27;19(12):e0315245. doi: 10.1371/journal.pone.0315245 (PMC11676921; doi:10.1371/journal.pone.0315245)
Supplement: Attachment — Submitted filename: Response to Reviewers.docx [file pone.0315245.s003.docx]

**Response to Reviewers’ comments**

**Reviewer #1:**

The manuscript entitled “Discovery of Novel TACE Inhibitors Using Graph Convolutional Network, Molecular Docking, Molecular Dynamics Simulation, and Biological Evaluation” has many mistakes, authors need to rectify many portions.

• Line 97: The sentence "Therefore, its potential to identify novel TACE inhibitors with greater accuracy and efficiency was examined in this study" is unclear. The sentence does not specify what "its" refers to. It should be rewritten for clarity.

The suggested line has been rewritten for clarity and readability.

• Line 79: The phrase "TNF-α, is also produced by neurons and glia," includes an unnecessary comma after "TNF-α."

The Typo has been corrected.

• Line 191: The sentence "TACE, Ligand, and Zn2+ ion was uploaded to the server for each complex and CHARMM36 force field [32] setup was performed using the solution builder protocol on the CHAR..." ends abruptly and seems incomplete.

The line has been rephrased for better understanding.

• The author required to update recent references can be seen PMID: 37065061, 39065802, 35424125, 34297427, 35014595, 36936534.

All the suggested citations have been cited in the manuscript.

• Check the sentence: "metrics such as the Area Under the Curve (AUC) of the Receiver Operating Characteristic (ROC) curve..."

The sentence has been rephrased.

• Confusing sentence please correct it: TPR of 1 usually indicates perfect performance at a specific threshold, but it doesn’t make sense in relation to the ROC curve unless further clarification is provided.

The suggested sentences have been rewritten for clarity and readability.

• It's unclear whether "perfect predictions" refers to all metrics being 1.0 or something else. A more precise explanation would enhance clarity.

A precise elaboration has been provided for the clarity.

• "2OI0 and co-crystallized ligand was utilized to figure out the interacting amino acid residues..."

The sentence has been rewritten for clarity.

• Check the sentence "Quinapril, Vorinostat, and Alvimopan with their exceptionally low CDocker energy, emerge as a promising candidate..."

The sentence has been rewritten for clarity and readability.

We would like to thank you again for your careful reading and critical comments on our manuscript. We revised the manuscript based on your comments. We appreciate that your comments and suggestions significantly enhanced the quality of our work.

**Reviewer #2:**

The authors work on Discovery of Novel TACE Inhibitors Using Graph Convolutional Network, Molecular Docking, Molecular Dynamics Simulation, and Biological Evaluation contributes to academic knowledge, but the discussion need to be improved upon. Further comments can be found on the annotated report

Line 108: The accession date has been updated

Line 118: The line has been rephrased according to the suggestion

Line 120: The typo mistake has been corrected.

Line 130: The deep learning architecture from our previous publications and other publications has been discussed and cited in the manuscript.

Line 140: The lines has been rephrased

Line 144: The citation from our previous study has been cited.

Line 172: TACE is Metalloproteinase which requires Zn2+ at the site of binding for stable interaction. The information is updated.

Line 268: We appreciate the reviewer’s insightful comment regarding the significance of the observed distribution of molecular descriptor values between active and decoy compounds. To address this concern, we conducted additional statistical analysis, calculating the mean ± standard deviation for the descriptors in both groups. The results confirm that there are no significant differences between the active and decoy groups for the evaluated descriptors. This observation is visually supported by the attached figure, which clearly illustrates the comparable distribution of values (Please see the attached figure below).

Line 322: The Typo has been corrected

Line 590: A brief discussion has been added in comparison with previous studies validating the computational methodologies.

We would like to thank you again for your careful reading and critical comments on our manuscript. We revised the manuscript based on your comments. We appreciate that your comments and suggestions significantly enhanced the quality of our work.
